# Five Years of COVID-19 in Tocantins, Brazil: Epidemiology, Vaccination Impact, and SARS-CoV-2 Genomic Dynamics (2020–2025)

**DOI:** 10.3390/v17111521

**Published:** 2025-11-20

**Authors:** Olivia de Souza da Conceição, Ueric José Borges de Souza, Franciano Dias Pereira Cardoso, Evgeni Evgeniev Gabev, Bergmann Morais Ribeiro, Gil Rodrigues dos Santos, Renisson Neponuceno de Araújo Filho, Marcos Gontijo da Silva, Fernando Rosado Spilki, Fabrício Souza Campos

**Affiliations:** 1 Bioinformatics and Biotechnology Laboratory, Campus of Gurupi, Federal University of Tocantins, Gurupi 77410-570, TO, Brazil; souza.olivia@mail.uft.edu.br; 2 Central Public Health Laboratory of the State of Tocantins, Palmas 77054-970, TO, Brazil; francianocardoso@yahoo.com.br; 3 Department of Physiology and Pathophysiology, Medical University of Sofia, 1431 Sofia, Bulgaria; egabev@medfac.mu-sofia.bg; 4 Baculovirus Laboratory, Department of Cell Biology, Institute of Biological Sciences, University of Brasilia, Brasília 70910-900, DF, Brazil; bergmann.ribeiro@gmail.com; 5 Laboratory of Phytopathology, Campus of Gurupi, Federal University of Tocantins, Gurupi 77402-970, TO, Brazil; gilrsan@mail.uft.edu.br; 6 Departamento de Tecnologia Rural, Universidade Federal Rural de Pernambuco, Recife 52171-900, PE, Brazil; renisson.neponuceno@ufrpe.br; 7 Programa de Pós-Graduação em Biotecnologia, Campus of Gurupi, Universidade Federal do Tocantins, Gurupi 77402-970, TO, Brazil; gontijobio@uft.edu.br; 8 Molecular Microbiology Laboratory, Health Sciences Institute, Feevale University, Novo Hamburgo 93525-075, RS, Brazil; fernandors@feevale.br; 9 Laboratório de Bioinformática & Biotecnologia, Instituto de Ciências Básicas da Saúde, Universidade Federal do Rio Grande do Sul, Porto Alegre 90010-150, RS, Brazil

**Keywords:** COVID-19, SARS-CoV-2, vaccination impact, genomic surveillance

## Abstract

The coronavirus disease 2019 (COVID-19) pandemic in Tocantins, Brazil, exhibited distinct phases between 2020 and 2025, with high mortality concentrated in 2020–2021 and subsequent stabilization at residual levels. Using epidemiological data, statistical modeling, and genomic surveillance, we show that the crisis peaked in 2021, coinciding with the circulation of Gamma and Delta, when health system capacity was severely strained. From 2022 onwards, the spread of Omicron led to record incidence but proportionally low mortality, reflecting accumulated immunity, vaccination, and improved clinical management. Vaccination represented the turning point, reducing hospitalizations and deaths by over 90% and driving a clear decoupling between incidence and severity. Interrupted time-series and generalized additive model (GAM) analyses confirmed sustained reductions in transmission and severity associated with mass immunization. Genomic sequencing of 3941 severe acute respiratory syndrome coronavirus 2 (SARS-CoV-2) genomes identified 166 lineages and successive variant replacements, culminating in the predominance of LP.8.1.4 in 2025. To our knowledge, this is one of the few integrated, long-term analyses (2020–2025) combining epidemiological and genomic data, capturing the full succession of variants up to LP.8.1.4 and highlighting Tocantins as a strategic “variant corridor” linking Brazil’s North and Central-West regions. These findings underscore the dual role of vaccination and genomic surveillance in shaping the epidemic trajectory and the importance of sustaining both strategies to mitigate future health crises.

## 1. Introduction

The emergence of severe acute respiratory syndrome coronavirus 2 (SARS-CoV-2) in December 2019 in Wuhan, China, triggered an unprecedented global health crisis, resulting in millions of cases and deaths within the first months [1]. By mid-2025, more than 775 million cases and approximately 7 million deaths had been reported, highlighting the profound impacts on public health, the economy, and social structures [2]. In Brazil, the first case was confirmed in São Paulo in February 2020. The virus spread rapidly, with community transmission soon recognized nationwide. In the state of Tocantins, the first case was recorded in the capital city of Palmas on 18 March 2020. From there the virus disseminated along major mobility corridors, with strategic high-population-density municipalities acting as central hubs for viral entry and dispersion into smaller towns and rural areas [3,4].

Belonging to the genus *Betacoronavirus* and subgenus *Sarbecovirus*, SARS-CoV-2 has a large RNA genome of approximately 30,000 nucleotides that encodes essential structural proteins. These include the spike (S) protein, which binds to the human angiotensin-converting enzyme 2 (ACE2) receptor, predominantly on respiratory epithelial cells, to mediate viral entry and define its high infectivity [5,6,7,8]. The envelope (E) and membrane (M) proteins are associated with virion assembly and structural stability, respectively, while the nucleocapsid (N) protein packages the viral RNA and aids in replication regulation [1,9]. Crucially, despite the action of the nsp14 exonuclease error-correcting mechanism, the virus’s replication fidelity allows for sufficient replication error rates. This inherent capacity for genetic variation is a fundamental driver of its diversity and ongoing evolution [10].

The field of viral genomics relies on precise terminology to track viral evolution. A lineage is a group of viruses with a common genetic ancestor and a characteristic mutation set, used primarily for tracing dissemination. When a lineage accumulates mutations that have meaningful functional consequences, such as enhanced transmissibility, increased virulence, or immune escape, it is termed a variant [1,11]. To assess public health risk, the World Health Organization (WHO) classifies such variants as variants of interest (VOI) or, for those with demonstrated significant epidemiological impact, variants of concern (VOC) [2,12]. Major VOCs like Alpha, Beta, Gamma, Delta, and Omicron each dramatically reshaped the pandemic dynamics by exploiting these adaptive advantages [10,13,14,15,16,17].

In the state of Tocantins, the spread of coronavirus disease 2019 (COVID-19) was markedly influenced by regional factors, including significant demographic disparities, major transportation corridors like the BR-153 highway (also called Belém–Brasília highway), and unequal access to healthcare. Consequently, the burden of disease was heaviest in areas of high population density and mobility, with vulnerable populations, such as indigenous, quilombola, and riverside communities, disproportionately affected [18,19,20,21,22,23]. Although the international public health emergency was declared over in May 2023, the continuous emergence of new SARS-CoV-2 sublineages underscores the critical need for sustained, integrated genomic and epidemiologic surveillance to monitor the virus’s ongoing impact [2].

Previous studies have established a crucial foundation for understanding the COVID-19 pandemic in Tocantins. Epidemiological research has detailed the outbreak’s burden, noting the heightened severity of the second wave [24], and mapped the initial spatial concentration of cases in the capital, Palmas [25]. Complementing this, genomic surveillance efforts have documented the specific circulation of variants, from the P.1.7 sublineage to the later dominance of Omicron sublineages like XBB.1.18.1 [10,23]. At the national level, a recent analysis by Souza et al. [10] contextualized these efforts, revealing major disparities in genomic surveillance across Brazil. Their work highlighted that while Tocantins did not sequence the highest absolute number of genomes, its proportional sequencing rate was among the nation’s highest, indicating a robust and proactive monitoring program [10].

Specifically, this study aimed to quantify the impact of vaccination and variant dynamics on COVID-19 outcomes in Tocantins between 2020 and 2025 through integrated epidemiological, statistical, and genomic analyses. Therefore, this study provides an integrated, longitudinal analysis of the epidemiological, sociodemographic, and genomic evolution of COVID-19 in Tocantins from March 2020 to May 2025. By correlating the introduction and dominance of key variants (Gamma, Delta, Omicron, and their sublineages) with local indicators such as incidence, mortality, hospitalizations, and vaccination coverage, we offer an unprecedented overview of the pandemic’s waves in the state. Importantly, Tocantins emerges not merely as a recipient but as a dynamic corridor for the circulation and dissemination of SARS-CoV-2 variants across Brazil’s North and Central-West regions. This perspective highlights how structural vulnerabilities, including socioeconomic inequality, interstate mobility, and evolving public policies, shaped the five-year trajectory of the pandemic. By consolidating epidemiological and genomic insights, this work provides a robust evidence base to guide more effective public health policies and strengthen genomic surveillance systems in Brazil and beyond.

## 2. Materials and Methods

### 2.1. Source Data

Epidemiological data on confirmed COVID-19 cases, deaths, hospital admissions, and vaccination coverage were obtained from the official health surveillance platform of the state of Tocantins, Integra Saúde Tocantins (https://integra.saude.to.gov.br/covid19, accessed on 21 August 2025). Data were aggregated by epidemiological week according to the Brazilian national reporting system. The first week of 2020 was defined as 2–4 January, with subsequent weeks starting on Sundays. This definition ensured consistency with national public health reporting standards while aligning with the structure of the available dataset.

To reduce short-term fluctuations and emphasize underlying temporal patterns, 7-day centered moving averages (MM7) were calculated for confirmed cases, deaths, hospitalizations, and vaccine doses. The smoothed time series were subsequently aggregated by epidemiological week and calendar year, yielding weekly totals and averages. From these aggregated data, key epidemiological indicators were derived. The incidence rate was defined as the number of confirmed cases per 100,000 inhabitants, assuming an estimated population of 1.6 million for the state of Tocantins. The mortality rate was expressed as the number of deaths per 100,000 inhabitants. Vaccination coverage was estimated as the cumulative number of doses administered divided by the total population, and the vaccine-to-case ratio was calculated as the number of doses administered per confirmed case, reflecting the intensity of immunization efforts relative to disease burden. All indicators were computed in R version 4.5.1 [26] and analyzed across distinct epidemiological phases to evaluate temporal changes in disease burden and public health response.

To assess the temporal impact of COVID-19 vaccination on epidemiological indicators, the study period was divided into three phases: (i) the pre-vaccination period (up to epidemiological week 2 of 2021); (ii) the initial vaccination campaign (weeks 3–26 of 2021), corresponding to the early rollout and prioritization of high-risk groups; and (iii) the widespread vaccination phase (week 27 of 2021 onward), reflecting broader population-level coverage. For each phase, weekly data were aggregated and used to assess changes in key indicators, including confirmed cases, deaths, hospitalizations, incidence, mortality, vaccination coverage, and the vaccine-to-case ratio. One-way analysis of variance (ANOVA) was performed for each indicator to test for differences among the three vaccination periods. When ANOVA results were significant (*p* < 0.05), Tukey’s Honest Significant Difference (HSD) post hoc test was applied to determine pairwise differences between periods. All statistical analyses were conducted in R (version 4.5.1), and visualizations were generated using the ggplot2 package [27].

### 2.2. Generalized Additive Models for Vaccination Impact Assessment

To investigate the non-linear relationships between weekly COVID-19 vaccine administration and key epidemiological outcomes, we fitted generalized additive models (GAMs) using restricted maximum likelihood (REML) estimation. This flexible modeling framework allows for the identification of complex associations between predictors and outcomes without imposing strict parametric assumptions.

Separate GAMs were developed for each outcome, smoothed using a seven-week moving average: confirmed cases, deaths, hospitalizations, incidence, mortality, vaccination coverage, and the vaccine-to-case ratio. Each model included three terms: (i) a smooth function of time elapsed since the start of the series (in weeks); (ii) a cyclic smooth for epidemiological week of the year (52 levels) to capture recurring seasonal patterns; and (iii) a smooth function of the weekly average number of vaccine doses administered. Cubic regression splines were applied to the smooths for time and vaccination, whereas epidemiological-week effect was modeled with a cyclic cubic spline with a fixed basis dimension of k = 52 (approximately one basis function per week). Smoothing parameters were estimated by REML, and basis dimensions for the non-seasonal smooths were intentionally kept low (mgcv default k values) to balance flexibility and overfitting.

The significance of smooth terms was evaluated using F-statistics and estimated degrees of freedom (edf), while model performance was assessed by the adjusted coefficient of determination (R^2^) and the percentage of deviance explained. Assumptions of residual independence and homoscedasticity were verified for all model fits. Model validity over time was evaluated using Pearson residuals and their autocorrelation and partial autocorrelation functions. For all outcomes, residuals showed mild positive autocorrelation at short lags (approximately up to 4–6 weeks), which is expected for weekly surveillance series; therefore, GAM results are interpreted primarily as descriptive patterns of association rather than as fully independent observations. Concurvity diagnostics from the mgcv package indicated moderate concurvity between the smooths of calendar time and vaccination (estimate values around 0.4–0.5), reflecting their shared temporal structure but remaining well below 1, suggesting that the smooth terms remained identifiable. Partial effects of vaccine administration on each outcome were visualized as central estimates with ±1 standard error, allowing interpretation of the magnitude and direction of associations across the observed range of vaccine coverage. All models were implemented in the mgcv package in R (version 4.5.1), and partial-effect plots were generated with ggplot2 to depict the non-linear relationships between vaccination and epidemiological outcomes, accounting for both temporal and seasonal components.

### 2.3. Time Series Analysis of Epidemiological Indicators

To evaluate the temporal impact of the COVID-19 vaccination campaign on key epidemiological indicators in Tocantins, we applied interrupted time-series (ITS) analysis using segmented regression models, a standard approach for assessing intervention effects in epidemiological time series. Weekly smoothed values (7-week moving averages, MM7) of confirmed cases, deaths, hospitalizations, incidence, and mortality were modeled independently. The intervention point was set at the third epidemiological week of 2021 (17 January), marking the official start of the vaccination campaign in the state.

For each outcome variable ($Y_{t}$) the model was specified as follows:

$$Y_{t}=\beta_{0}+\;\beta_{1\;}\times \;{Time}_{t}+\;\beta_{2}\;\times \;Post_{t}+\beta_{3}\;\times \;{TimePost}_{t}\;+\;\varepsilon_{t}$$

where ${Time}_{t}$ is a continuous variable indicating the week index; $Post_{t}$ is a binary variable indicating whether week *t* occurred after the intervention (0 = before, 1 = after); ${TimePost}_{t}$ counts the number of weeks since the intervention; $\beta_{0}$ estimates the intercept (baseline level) at the beginning of the series; $\beta_{1}$ estimates the pre-intervention slope; $\beta_{2}$ estimates the immediate change in level after the intervention; $\beta_{3}$ estimates the change in slope post-intervention, and $\varepsilon_{t}$ is the residual error.

Models were fitted using ordinary least squares (OLS) with heteroskedasticity-robust standard errors (HC3). Serial correlation was assessed by visual inspection of residual time series and their autocorrelation and partial autocorrelation functions. Residuals exhibited the expected pattern of short-lag positive autocorrelation for weekly surveillance data; accordingly, the ITS coefficients were interpreted as descriptive summaries of changes in level and trend around the vaccination onset, rather than as estimates based on fully independent observations.

To further disentangle long-term trends, recurrent seasonal patterns and irregular fluctuations in the COVID-19 epidemiological indicators, we applied seasonal-trend decomposition using loess (STL), originally introduced by Cleveland et al. (1990) [28]. This robust, nonparametric method decomposes time series into three additive components—trend, seasonality, and remainder—while maintaining reliability in the presence of outliers or mild nonstationarity.

Weekly smoothed values (7-week moving averages) were calculated for confirmed cases, deaths, hospitalizations, incidence, mortality, and vaccination coverage to minimize short-term noise and enhance interpretability. All series were aligned with ISO-8601 calendar weeks [29]. STL decomposition assumed an annual seasonal cycle of 52 weeks. To reduce sensitivity to anomalous observations, robust fitting procedures and periodic seasonal windows were applied. Time series were carefully indexed to ensure temporal consistency, and each component—trend, seasonality, and remainder—was visualized using the ggplot2 package to facilitate interpretation. All analyses were performed in R (version 4.5.1).

### 2.4. Genomic Analysis

SARS-CoV-2 genome sequences and associated metadata were retrieved from the global initiative on sharing all influenza data (GISAID; https://gisaid.org) on 30 June 2025 [30]. Analyses were restricted to samples collected in the state of Tocantins, Brazil, as indicated in the “Location” field in GISAID at the first administrative level (“Brazil/Tocantins”). Records geocoded only at the country level or presenting ambiguous/conflicting location information were excluded from the dataset. We gratefully acknowledge the originating and submitting laboratories for making these data publicly available through GISAID.

In this study we analyzed the consensus genomes as made available in GISAID and did not process raw sequencing reads. To minimize the impact of low sequencing depth and assembly artifacts, we applied metadata-based quality filters: only genomes flagged by GISAID as complete and high coverage were retained, and sequences with very short length or excessive ambiguous bases (high N content) were excluded. After this filtering, only sequences assigned to “Brazil/Tocantins” were retained for downstream analyses.

SARS-CoV-2 lineages were assigned using pangolin with the pango-designation database current as of 30 June 2025 [31]. For interpretability, assigned lineages were grouped by prefix into “families” (e.g., JN, KP, LP, XBB), with remaining categories aggregated as “other”. To enable comparisons across epidemiological phases, results were summarized across two-time windows: January 2020–December 2022 and January 2023–June 2025. Within each window, sequences were aggregated by collection month and lineage. For each aggregation, we report (a) monthly counts and (b) monthly relative frequencies, computed after completing the full month × lineage grid and renormalizing to sum to 100% per month. This normalization helped attenuate the impact of variable sequencing throughput over time and between epidemic waves. All figures were generated in R (version 4.5.1) using the ggplot2 package.

As a plausibility check for lineage assignment, we visually inspected the temporal patterns of major lineages (e.g., Gamma, Delta, Omicron sublineages and LP.8.1/PD families) and to confirm that their emergence and replacement were consistent with national and global SARS-CoV-2 circulation patterns and with the expected chronological order of variants.

### 2.5. Phylogenetic Analysis

To complement the genomic frequency analyses, we performed phylogenetic reconstruction using the Nextstrain SARS-CoV-2 workflow (augur/auspice) [32]. This approach allowed us to contextualize the Tocantins genomes within a global phylogenetic framework, employing the Nextstrain Open background dataset (global metadata and sequences; accessed on 30 June 2025: metadata: https://data.nextstrain.org/files/ncov/open/global/metadata.tsv.xz, sequences: https://data.nextstrain.org/files/ncov/open/global/sequences.fasta.xz). Sequence alignment was carried out with *Nextalign* [33] against the Wuhan-Hu-1 reference genome (MN908947.3), applying Nextstrain’s standard masking of terminal regions and community-curated problematic sites. Global contextualization followed Nextstrain’s subsampling strategy (country/month quotas with diversity retention), with all Tocantins genomes forcibly retained in the dataset. A maximum-likelihood phylogeny was inferred within the pipeline and subsequently converted into a time-scaled tree using TreeTime [34] under the standard SARS-CoV-2 molecular clock prior. The resulting trees and associated metadata summaries were visualized and explored through the Auspice interface (Nextstrain).

## 3. Results

### 3.1. Epidemic Dynamics and Vaccination Rollout in Tocantins

**Figure 1:**
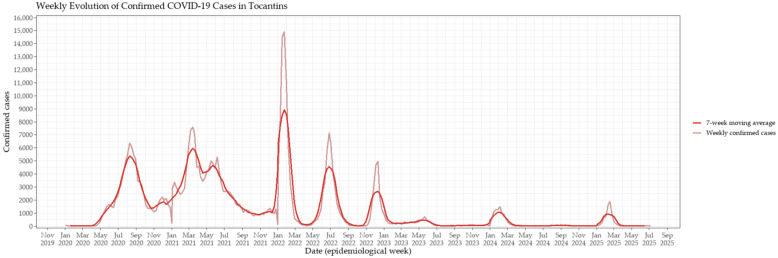
Weekly evolution of confirmed COVID-19 cases in Tocantins, Brazil. The time series displays the number of confirmed cases reported weekly from early 2020 to mid-2025. Distinct peaks correspond to major epidemic waves, notably those associated with the Gamma variant (early 2021) and the Omicron variant (early 2022). The red line depicts the 7-week moving average, applied to smooth short-term fluctuations and emphasize underlying long-term trends.

The epidemic trajectory of COVID-19 in Tocantins followed a multiphasic pattern, with successive waves of infection and mortality shaping the course of the pandemic. Weekly confirmed case counts were initially low during the early months of 2020 but began to rise steadily by midyear, culminating in the first major wave between July and September 2020 (Figure 1). A second, more severe wave occurred in early 2021, with sustained high transmission levels between March and May. The most pronounced surge occurred in early 2022 during the Omicron wave; however, mortality remained comparatively lower, a decoupling between incidence and deaths that marked the post-vaccination period. The seven-week moving average clearly delineated the onset, peak, and decline of each wave, providing a smoothed representation of their temporal dynamics (Figure 1).

**Figure 2:**
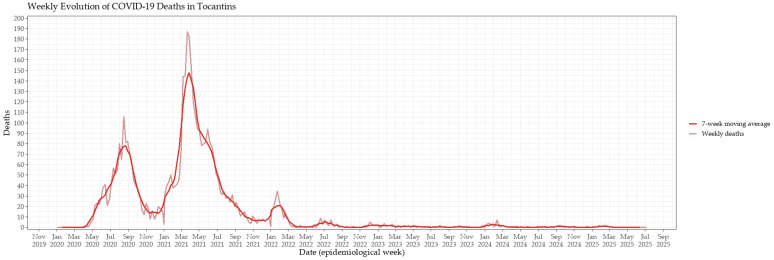
Weekly evolution of COVID-19–related deaths in Tocantins, Brazil, from 2020 to mid-2025. The light red line represents weekly reported deaths, while the dark red line depicts the 7-week moving average, applied to smooth short-term fluctuations and highlight the temporal dynamics of mortality peaks.

Weekly COVID-19–related deaths exhibited a similar temporal trend to confirmed cases, though with a slight delay relative to case peaks, consistent with the clinical progression of severe disease (Figure 2). The first mortality peak occurred in August 2020, followed by a sharper and more prolonged peak in April 2021. Notably, while confirmed cases reached their maximum during the 2022 wave, the corresponding rise in weekly deaths was proportionally smaller compared with earlier waves. This decoupling between incidence and mortality in the later stages of the pandemic likely reflects the combined effects of accumulated natural and vaccine-induced immunity, improved access to healthcare, expanded diagnostic capacity, and advances in the clinical management of severe cases.

**Figure 3:**
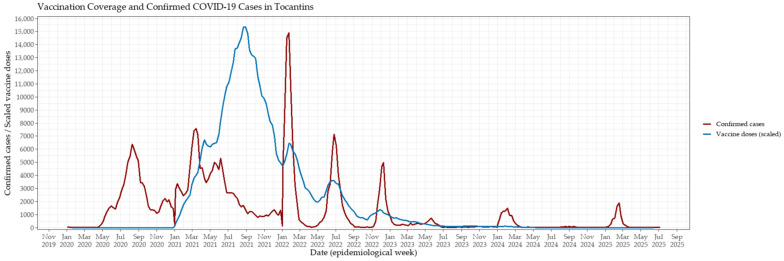
Weekly evolution of confirmed COVID-19 cases and vaccination coverage in Tocantins, Brazil, from 2020 to mid-2025. The red line represents weekly confirmed cases, while the blue line indicates the number of vaccine doses administered per week, scaled by a factor of 5 to facilitate visual comparison. Peaks in vaccination coincided with subsequent declines in case counts, suggesting that increased coverage contributed to mitigating transmission and reducing incidence.

The COVID-19 vaccination campaign in Tocantins began in early 2021, with a rapid escalation in weekly vaccine administration observed throughout the first semester (Figure 3). Vaccination coverage peaked between May and November 2021, coinciding with the broadening of eligibility criteria and the intensification of statewide immunization efforts. This period was followed by a marked decline in both the incidence of confirmed cases and COVID-19–related mortality, suggesting a substantial epidemiological impact of mass vaccination on viral transmission and disease severity.

Notably, during the large surge of infections in early 2022—primarily driven by the Omicron variant—a clear decoupling between case incidence and mortality became evident. Despite record numbers of confirmed infections, the mortality burden was substantially lower than in previous waves. This divergence likely reflects enhanced population-level immunity resulting from widespread vaccination combined with prior SARS-CoV-2 exposure, underscoring the pivotal role of immunization in mitigating severe clinical outcomes.

Age-specific patterns revealed a clear decoupling between exposure and clinical severity. Most confirmed cases occurred among young and middle-aged adults, particularly those aged 30–39 years (83,844 cases), followed by 40–49 years (74,571) and 20–29 years (72,390). In contrast, children 0–9 years (19,464) and adults ≥80 years (8604) accounted for a relatively small proportion of diagnoses. Mortality patterns was inverted: deaths increased steeply with age, with only 21 deaths in children 0–9 years and 13 in adolescents 10–19 years, but a marked concentration among older adults, especially those aged 70–79 years (1015 deaths) and ≥80 years (1137 deaths). These patterns indicate that working-age adults drove transmission, whereas the greatest mortality burden fell on elderly groups (Figure S1A,B).

Sex-stratified indicators showed that women accounted for a larger share of diagnosed cases (56%), whereas men accounted for most deaths (59%). This pattern is consistent with higher health-seeking and testing behavior among women, and greater prevalence of comorbidities and lower adherence to preventive measures and vaccination among men. These gradients attenuated after widespread vaccination.

### 3.2. Comparative Analysis Across Vaccination Phases

COVID-19 epidemiological indicators varied significantly across the three vaccination periods defined in Tocantins, Brazil: the pre-vaccination (up to epidemiological week 2 of 2021), the initial vaccination campaign (weeks 3–26 of 2021), and the widespread vaccination phase (week 27 of 2021 onward). A one-way analysis of variance (ANOVA) was performed for each outcome, and when significant differences were detected, Tukey’s Honest Significant Difference (HSD) test was applied to identify pairwise contrasts (Table 1; Figure S2A–G).

Weekly confirmed cases differed significantly across vaccination periods (F(2, 290) = 35.5, *p* < 0.001). Average weekly case counts increased from 1709 in the pre-vaccination phase to 3921 during the initial campaign, before declining to 848 in the widespread vaccination phase. Tukey’s post hoc tests confirmed that all three periods differed significantly from one another (*p* < 0.05), with the highest burden observed during the initial campaign (Table 1; Figure S2A).

Weekly COVID-19–related deaths also varied significantly across vaccination periods (F(2, 290) = 202.0, *p* < 0.001). Mean weekly deaths rose from 24.6 in the pre-vaccination phase to 77.0 during the initial campaign, then dropped markedly to 3.01 in the widespread vaccination phase. All pairwise contrasts were highly significant (*p* < 0.001), underscoring the strong temporal association between vaccine rollout and reduced mortality (Table 1; Figure S2B).

Hospitalizations exhibited a comparable trajectory, with significant differences detected across phases (F(2, 290) = 252.0, *p* < 0.001) (Table 1; Figure S2C). Mean weekly hospital admissions increased from 47.3 in the pre-vaccination period to 133.0 during the initial campaign, then declined to 7.3 in the widespread vaccination phase. Tukey’s test confirmed significant differences among all three periods (*p* < 0.001).

Incidence rates also differed significantly across vaccination periods (F(2, 290) = 35.5, *p* < 0.001). Post hoc comparisons showed that incidence peaked during the initial campaign, significantly exceeding both the pre-vaccination (*p* < 0.001) and widespread vaccination periods (*p* < 0.001) (Table 1; Figure S2D). Mortality rates mirrored this trend (F(2, 290) = 202.1, *p* < 0.001), reaching their highest levels during the initial campaign before showing a pronounced decrease in the widespread vaccination phase (Table 1; Figure S2E).

Vaccination coverage increased markedly over time (F(2, 290) = 1564.0, *p* < 0.001), with significant differences detected between all three phases (Table 1; Figure S2F). The vaccine-to-case ratio also varied significantly (F(2, 290) = 8.11, *p* < 0.001), reaching its highest levels during the widespread vaccination period (*p* = 0.0005 vs. pre-vaccination) (Table 1; Figure S2G).

### 3.3. Non-Linear Effects of Vaccination Modeled Using GAMs

All fitted GAMs revealed a statistically significant association between weekly COVID-19 vaccine administration and each epidemiological indicator (*p* < 0.001 for all vaccination smooth terms; Table 2). The model for confirmed cases explained 81.1% of the deviance (adjusted R^2^ = 0.796), with an estimated degrees of freedom (EDF) of 4.66 for the vaccination spline. For deaths, model performance improved, with 89.5% of the deviance explained (adjusted R^2^ = 0.887, EDF = 4.77). The strongest fit was observed for hospitalizations, which showed 91.4% of the deviance explained (adjusted R^2^ = 0.907, EDF = 5.09) (Table 2).

Slightly lower, though still statistically robust, fits were obtained for incidence and mortality rates, with adjusted R^2^ values of 0.615 and 0.822, respectively (Table 2). The vaccine-to-case ratio model showed moderate explanatory power (adjusted R^2^ = 0.524), consistent with the higher variability inherent to this composite metric. As expected, vaccination coverage achieved a perfect fit (R^2^ = 1.0) due to its cumulative, monotonically increasing trajectory (Table 2). All GAMs incorporated smooth terms for long-term temporal trends (weeks) and seasonal variation (ISO week), ensuring reliable estimation of non-linear associations across the study period.

The estimated smooth functions revealed distinct non-linear associations between weekly vaccine doses and COVID-19 epidemiological indicators in Tocantins (Figure S3A–G). For confirmed cases, the partial effect initially increased with rising vaccination levels, peaking at approximately 30,000 weekly doses before declining sharply, likely reflecting improved case detection during the early rollout, followed by reduced transmission as population coverage expanded (Figure S3A). By contrast, deaths and hospitalizations exhibited pronounced and sustained declines beyond roughly 25,000 doses per week, consistent with the protective effect of vaccination against severe outcomes (Figure S3B,C).

Similar inverse relationships were observed for incidence and mortality rates, reinforcing the role of widespread immunization in reducing overall disease burden (Figure S3D,E). As expected, the vaccination-coverage curve displayed a cumulative pattern, whereas the vaccine-to-case ratio showed a steady upward trajectory, reflecting increasing population-level immunity relative to reported infections (Figure S3F,G). All partial-effect plots were adjusted for temporal and seasonal trends, with 95% confidence intervals displayed.

Collectively, these results highlight the non-linear, dose–response nature of vaccination effects across multiple epidemiological dimensions and suggest that, beyond a critical threshold, additional weekly doses yield diminishing marginal benefits.

### 3.4. Interrupted Time Series Analysis of Vaccination Impact

Before interpreting the segmented regression results, model validity was assessed through inspection of residual autocorrelation and partial autocorrelation functions. Residuals exhibited mild short-lag correlation typical of weekly surveillance data but showed no strong higher-order structure, supporting the descriptive use of the ITS framework. Robust (HC3) standard errors were applied to account for heteroskedasticity, and the smoothed (MM7) series helped reduce potential overdispersion and short-term seasonality.

**Figure 4:**
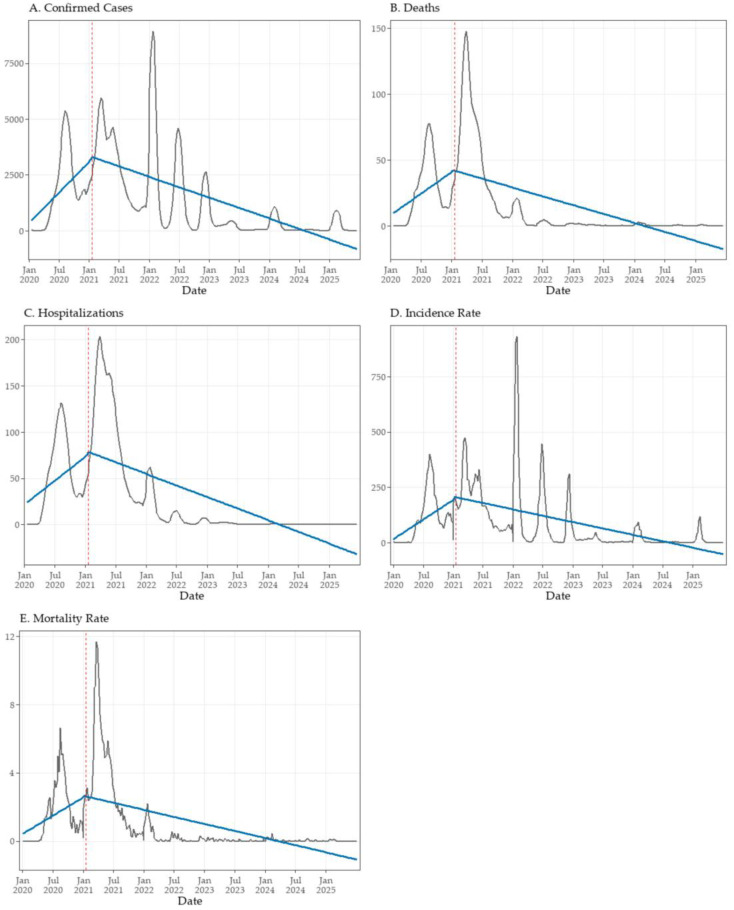
Interrupted time series (ITS) analysis of key COVID-19 epidemiological indicators in Tocantins, Brazil. Weekly smoothed values (7-week moving averages) are shown as black lines, while fitted segmented regression lines appear in blue. The vertical dashed red line marks the intervention point (epidemiological week 3 of 2021), corresponding to the start of the state’s vaccination campaign. Panels show: (**A**) confirmed cases, (**B**) deaths, (**C**) hospitalizations, (**D**) incidence rate, and (**E**) mortality rate.

These patterns were consistent with the gradual, cumulative effects captured by the GAM analyses, confirming a sustained post-vaccination decline across all indicators. The ITS models further quantified these effects, revealing consistent post-vaccination trend reversals across all analyzed COVID-19 indicators in Tocantins (Table 3; Figure 4A–E). Prior to the intervention, confirmed cases increased significantly on a weekly basis (β_1_ = +52.44; *p*  <  0.001) (Figure 4A). Although no immediate level change was detected at the onset of the vaccination campaign (β_2_ = +64.45; *p*  =  0.881), a significant post-intervention trend reversal was observed (β_3_ = −70.14; *p*  <  0.001), indicating a sustained and progressive decline in case counts.

Similarly, deaths (Figure 4B) and hospitalizations (Figure 4C) displayed significant upward trends prior to the intervention (β_1_ = +0.62 and +0.98; both *p*  <  0.001), followed by marked reversals in slope after vaccination began (β_3_ = −0.88 and −1.46; both *p*  <  0.001). As with confirmed cases, neither outcome exhibited an immediate level shift, indicating that the impact of vaccination on severe outcomes was gradual rather than abrupt.

For incidence (Figure 4D) and mortality rates (Figure 4E), pre-intervention trends were likewise significantly positive (β_1_ = +3.40 and +0.04; both *p*  <  0.001). Immediate level changes were not statistically significant (β_2_ = +0.51 and −0.08), but both indicators showed pronounced post-intervention reversals in slope (β_3_ = −4.49 and −0.06; both *p*  <  0.001). Taken together, these findings indicate that the vaccination campaign was associated with sustained reductions in COVID-19 transmission and severity, even in the absence of abrupt level shifts.

### 3.5. Temporal Decomposition of Epidemiological Indicators (STL Analysis)

The STL decomposition of the epidemiological time series in Tocantins revealed distinct temporal dynamics across COVID-19 indicators (Figure S4A–F). Confirmed cases exhibited a pronounced seasonal component, with recurrent midyear peaks, and a long-term trend characterized by a steady increase until early 2022, followed by a sustained decline (Figure S4A). The series also displayed short-term irregular fluctuations, likely reflecting localized outbreaks or inconsistencies in case reporting during critical phases of the pandemic.

The trajectory of COVID-19–related deaths partially mirrored the trends observed for confirmed cases, albeit with a less pronounced seasonal component (Figure S4B). The long-term trend showed a steep rise in mortality through mid-2021, followed by a gradual and sustained decline. Irregular fluctuations were also present, suggesting episodic mortality surges potentially associated with health system strain, the circulation of more virulent variants, or delays in death registration during periods of high disease burden. Hospitalizations followed a similar pattern, peaking in early 2022 and subsequently declining (Figure S4C). Seasonal variation was evident, while short-term deviations likely reflected changes in admission practices, access to care, or localized service pressures.

The incidence rate displayed a rising trajectory during the early phase of the pandemic, gradually tapering over time (Figure S4D). Its seasonal component closely aligned with the pattern observed for confirmed cases, whereas short-term variability reflected transient fluctuations in transmission dynamics or shifts in surveillance coverage. In contrast, the mortality rate exhibited a smoother and more stable profile (Figure S4E). Its long-term trend increased during the initial stage of the pandemic, followed by a clear and sustained decline from 2022 onward. Seasonal variation was minimal, and only minor residual anomalies were detected, likely attributable to reporting delays or changes in population vulnerability.

Vaccination coverage displayed a distinctly regular pattern (Figure S4F). The trend component showed a continuous and sustained increase beginning in early 2021, reflecting the progressive rollout of the vaccination campaigns. In contrast to other indicators, this series exhibited negligible seasonal or irregular variation, underscoring the structured and consistent implementation of vaccine distribution across the state.

### 3.6. SARS-CoV-2 Genomic Surveillance and Lineage Dynamics

Between September 2020 and May 2025, a total of 3941 SARS-CoV-2 genomes were sequenced in Tocantins, of which 168 lacked geographic metadata. The remaining 3773 genomes were successfully assigned to municipalities across the state. Sequencing efforts were heavily concentrated in Palmas (1895 genomes), followed by Gurupi (262), Porto Nacional (210), Araguaína (151), and Paraíso do Tocantins (113) (Figure S5; Table S1). Additional municipalities, including Formoso do Araguaia (73), Miracema do Tocantins (65), Miranorte (53), Alvorada (42), and Dueré (39), contributed smaller but epidemiologically meaningful numbers (Figure S5; Table S1).

The spatial distribution, illustrated in the heatmap, reflects underlying demographic patterns, healthcare infrastructure, and the geographic localization of diagnostic and sequencing facilities in Tocantins (Figure S5). Palmas, the state capital, hosts major hospitals, public health laboratories, and research institutions, explaining its disproportionate share of genomic sampling. In contrast, smaller or remote municipalities were underrepresented, a pattern likely driven not only by lower population density but also by logistical constraints on sample transport and the lack of local diagnostic infrastructure—factors that collectively shaped the genomic surveillance landscape.

A total of 166 distinct SARS-CoV-2 lineages were identified among the 3941 genomes sequenced in Tocantins between September 2020 and May 2025. Of these, 53 lineages (31.9%) were detected only once, reflecting sporadic introductions or isolated transmission events. The most frequently observed lineages were BA.1.14.1 (797 genomes), XBB.1.18.1 (233), AY.99.2 (225), BQ.1.1 (223), and P.1 (151), which together accounted for a substantial portion of the dataset (Figure S6A).

By early 2025, the genomic landscape in Tocantins was shaped by the circulation of 19 distinct lineages between January and May (Figure S6B). A clear predominance of LP.8.1.4 (62 genomes) was observed, establishing this lineage as the dominant background during the period. In parallel, PD sublineages—notably PD.1 (17 genomes) and PD.1.2 (17 genomes)—emerged as relevant co-circulating variants, while LP.8.1.2 (9 genomes), PD.2 (4 genomes), and JN.1.11 (4 genomes) were detected at moderate frequencies. The remaining lineages, including JN.1.49.1, KP.2.3.12, LP.8.1.1, MC.33.1, KP.1, LF.7.5, LP.7, MC.10.1, NY.1, PD.2.1, XDQ, XEC, and XEC.32, appeared only sporadically (≤2 detections each), suggesting restricted local transmission or isolated introductions (Figure S6B).

At the municipal scale, lineage diversity closely reflected sequencing depth. Palmas, which contributed the largest number of genomes, also exhibited the broadest lineage spectrum (132 lineages), followed by Gurupi (57 lineages), Porto Nacional (39 lineages), Araguaína (38 lineages), and Paraíso do Tocantins (33 lineages) (Table S1). This pattern highlights the direct relationship between sequencing intensity and the ability to capture viral diversity: municipalities with higher sequencing volume revealed a broader array of circulating lineages, whereas those with limited sampling often showed only one or two variants, reducing epidemiological resolution.

**Figure 5:**
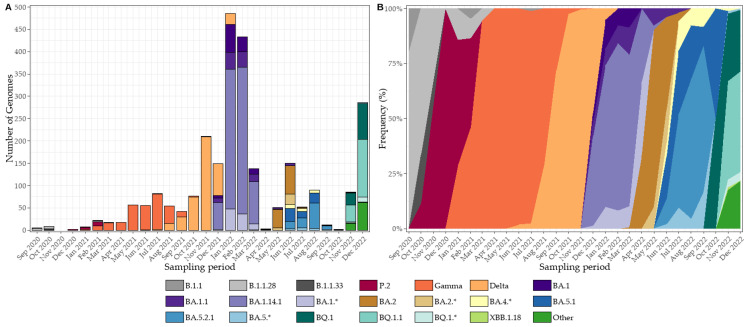
Monthly dynamics of SARS-CoV-2 lineages in Tocantins, Brazil (2020–2022). (**A**) Absolute counts of genomes sequenced per month, stratified by Pango lineage. Peaks in sequencing activity coincide with major epidemic waves, particularly during the Gamma wave and the onset of Omicron. (**B**) Relative monthly frequencies of lineages, normalized so that the sum of all lineages equals 100% in each month. The vertical thickness of each colored band represents the proportion of genomes assigned to that lineage in that month (stacked area plot; not confidence intervals or kernel bandwidths). Colors denote lineage families, with consistent hues applied to related sublineages. Normalized trajectories highlight successive shifts in viral dominance, independent of sequencing volume. * Lineages marked with an asterisk indicate groups of related Pango sublineages that were combined for visualization purposes due to low individual counts.

During 2020, SARS-CoV-2 transmission in Tocantins was dominated by B.1.1 lineages, particularly B.1.1.28 and B.1.1.33, with P.2 emerging toward the end of the year (Figure 5A,B). In early 2021, Gamma (P.1) expanded rapidly and maintained dominance throughout the first half of the year, before being progressively displaced by Delta in late 2021. The early months of 2022 were characterized by the Omicron BA.1 wave (including BA.1 and BA.1.14.1), followed by the co-circulation of BA.2 and BA.4, and subsequently by the rise of BA.5 (notably BA.5.1 and BA.5.2.1), reflecting successive within-Omicron lineage replacements (Figure 5A,B).

Sequencing throughput varied over time, with more genomes generated during epidemic peaks (particularly during the Gamma wave and the onset of Omicron) and fewer sequences processed between waves. This fluctuation is reflected in the count panels, where epidemic surges correspond to higher bars. In contrast, normalized frequency trajectories demonstrate that lineage turnover was not an artifact of sequencing volume but reflected genuine shifts in viral dominance: from early B.1.1 lineages to Gamma, followed by Delta, and ultimately successive Omicron sublineages.

**Figure 6:**
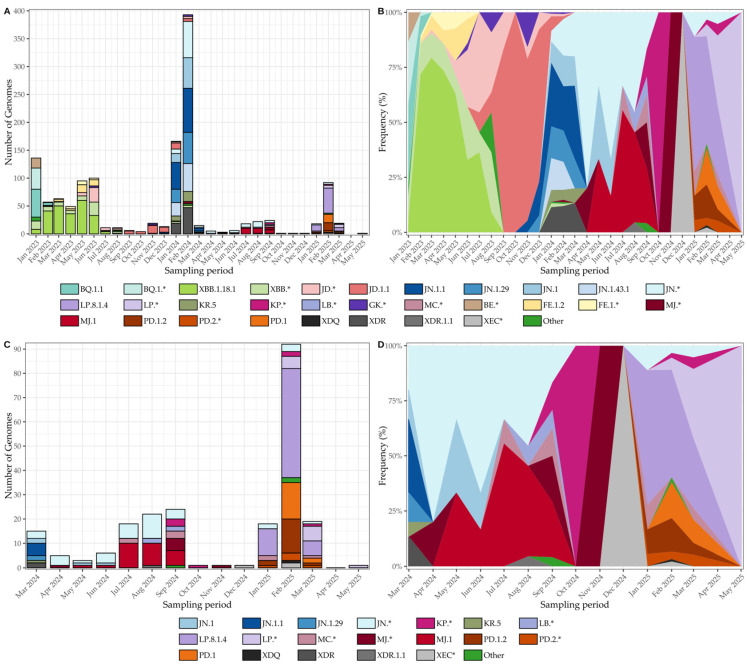
SARS-CoV-2 lineage dynamics in Tocantins, Brazil, from January 2023 to May 2025. (**A**) Monthly genome counts, illustrating fluctuations in sequencing throughput, with peaks coinciding with epidemic waves. (**B**) Normalized lineage frequencies for the same period, displayed as stacked area plots in which the bands sum to 100% per month. The vertical thickness of each colored band corresponds to the proportion of genomes assigned to each lineage, highlighting the orderly replacement of BQ/BE by XBB, JD, and ultimately JN. (**C**) Monthly genome counts from March 2024 to May 2025, emphasizing the expansion of JN sublineages and the emergence of MJ, KP, XEC, and LP/PD lineages. (**D**) Normalized lineage frequencies for March 2024–May 2025, again shown as stacked monthly proportions, revealing a coherent within-Omicron succession, from JN dominance to the rise of LP.8.1.4 and sustained PD activity, largely independent of changes in sequencing volume. * Lineages marked with an asterisk indicate groups of related Pango sublineages that were combined for visualization purposes due to low individual counts.

In early 2023, concurrent circulation of BQ.1/BQ.1.1 and BE lineages was documented, followed shortly by the emergence of XBB, with XBB.1.18.1 appearing as an early representative of this group (Figure 6A,B). By mid-2023, JD lineages (e.g., JD.1/JD.1.1) increased in prevalence, whereas GK appeared only sporadically, reflecting a gradual reshaping of the Omicron landscape. Toward the end of 2023, JN lineages, most notably JN.1.1 and JN.1.29, emerged and consolidated as the dominant background by March 2024. Although sequencing throughput fluctuated across months (Figure 6A), the normalized frequency trajectories (Figure 6B) reveal a smooth and orderly transition from BQ/BE to XBB/JD, and ultimately, to JN, largely unaffected by variability in sequencing intensity.

From March 2024 onward, JN and its sublineages (JN.1, JN.1.1, JN.1.29) predominated, with only sporadic detection of recombinants such as XDR. During April–May 2024, the MJ lineage (including MJ.1) emerged and briefly co-circulated with JN. By late 2024, KP was introduced at low-to-moderate levels, and XEC persisted sporadically (Figure 6C,D). A major transition occurred in early 2025, when LP.8.1.4 rose to predominance alongside sustained PD activity (PD.1, PD.1.2, PD.2*), coinciding with the decline of JN. Between March and June 2025, LP maintained dominance with intermittent PD and KP detections and residual traces of JN and MC. As in earlier periods, sequencing throughput varied (Figure 6C), yet normalized frequencies (Figure 6D) delineate a coherent within-Omicron succession, from JN dominance to the rise of LP.8.1.4 and persistent PD circulation, independent of sampling variability.

**Figure 7:**
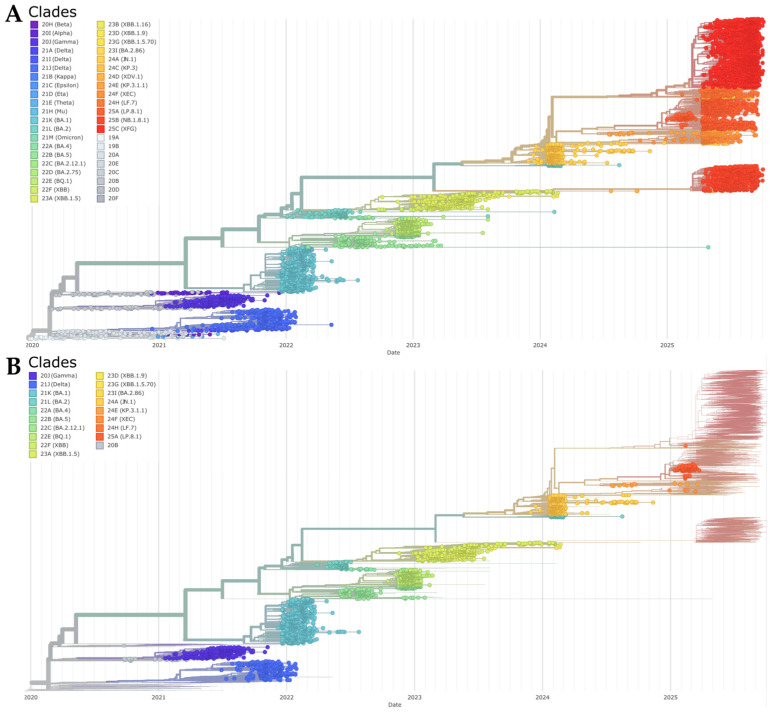
(**A**) Global time-scaled phylogeny of 8121 SARS-CoV-2 genomes, colored by Nextclade designation, illustrating sequential lineage turnover culminating in early 2025 with the predominance of clade 25A (LP.8.1/LP.8.1.4) and residual detection of 25B and 25C. (**B**) The same phylogeny with Tocantins sequences highlighted, showing their distribution across multiple clades, consistent with repeated introductions followed by localized spread (Auspice view, Nextstrain).

We reconstructed a time-scaled phylogeny of 8121 SARS-CoV-2 genomes using the Nextstrain workflow to contextualize Tocantins sequences within a global framework (Figure 7A). Tocantins genomes were distributed across multiple geographically diverse clades, rather than forming a single monophyletic cluster, indicating repeated introductions followed by local onward transmission (Figure 7B). Early lineages (2020–2021) clustered within pre-Omicron clades 20A/20B and the Gamma variant (20J). From late 2021 onward, Omicron lineages became dominant, with sequential replacements from 21K (BA.1) to 21L (BA.2) and 22B (BA.5) throughout 2022. During 2023, Tocantins sequences mirrored global trends, capturing the rise of 22E (BQ.1) and 23A/22F (XBB, XBB.1.5). The phylogeny reflects the progressive diversification of Omicron sublineages and their temporal succession in the state.

From late 2023 into 2024, the phylogeny revealed the emergence of clade 23I (BA.2.86/JN.1), followed by 24C (KP.3) and 24D (XDV.1), reflecting ongoing lineage turnover. In early 2025, clade 25A (LP.8.1), particularly LP.8.1.4, expanded to predominance, while residual signals of 25B (NB.1.8.1) and 25C (XFG) persisted at low levels. The short internal branches and stepwise turnover across calendar time are consistent with rapid lineage replacement within Omicron, mirroring global patterns while documenting repeated introductions and local diversification in Tocantins. Consistent with these dynamics, Nextstrain’s “emerging lineages” module flagged XBB.1.9.1, XBB.1.42, FE.1, JD.1, and LB.1 among Tocantins-associated sequences, as shown in the root-to-tip regression and time-scaled phylogeny (Figure S7).

## 4. Discussion

These findings provide the foundation for a broader interpretation of how epidemiological and genomic dynamics shaped the COVID-19 pandemic in Tocantins. Between 2020 and 2025, the epidemic unfolded in well-defined phases. The first two years were characterized by high mortality, particularly in 2021, when the case fatality rate reached 1.81%. This period coincided with the circulation of the Gamma (P.1) and Delta variants, both associated with increased clinical severity and substantial hospitalization demand [11,23,35], ultimately overwhelming the state health system. Our results confirm that this was the apex of the crisis, marked by hospital overload and pronounced inequalities in the response capacity across municipalities, consistent with the findings of Cesar et al. (2021) [24]. Similar patterns were documented nationally and globally during the Gamma and Delta waves, underscoring the broader relevance of the Tocantins context [9,12,23].

From early 2022 onwards, the epidemiological landscape shifted markedly. The emergence of the Omicron variant triggered record infection levels but was accompanied by a pronounced decline in mortality, with fatality rates falling to 0.19%. This decoupling between incidence and mortality reflects accumulated population immunity, stemming from widespread vaccination and prior infections, as well as advances in clinical management and improved health-system preparedness [9,10,12,16]. In Tocantins, the reduction was especially pronounced, indicating that vaccine coverage had reached protective thresholds even amid intense viral circulation.

As vaccination expanded and hospital capacity stabilized, COVID-19 transitioned into a phase of lower-severity endemic circulation. The time-series analyses revealed successive waves of differing intensity, shaped by both biological factors and determinants. The 2021 Gamma–Delta wave represented the peak of severity, whereas the 2022 Omicron wave marked the onset of a low-lethality endemic phase. This transition highlights the combined effects of immunization, improved clinical care, expanded bed availability, and strengthened preventive practices [36,37,38,39].

Spatial analysis revealed marked disparities across the state. Although Palmas and Araguaína registered the largest absolute numbers of cases and deaths, smaller municipalities exhibited disproportionately high early fatality rates, sometimes exceeding 5%. These patterns suggest not only greater severity among detected cases but also underreporting of mild infections and substantial barriers to diagnosis and hospitalization [4,25]. Such findings expose long-standing structural vulnerabilities in remote regions, consistent with broader evidence of social and health inequalities disproportionately affecting vulnerable populations [19,40,41].

Although socioeconomic and mobility indicators were not explicitly modeled, the heterogeneous patterns observed between large urban centers and smaller remote municipalities align with national evidence showing that income, urbanization, mobility patterns, and healthcare access shape COVID-19 transmission and outcomes [42,43,44]. In Tocantins, municipalities such as Palmas and Araguaína concentrate population, services, and intermunicipal mobility, which likely facilitated early introduction and sustained viral circulation, as reflected in higher incidence and greater lineage diversity. By contrast, remote municipalities with limited healthcare capacity and longer travel times to referral hospitals reported fewer confirmed cases but disproportionately high case-fatality rates, suggesting barriers to testing, delayed care, and structural deprivation [24,42,45]. These findings support the hypothesis that socioeconomic vulnerability and mobility flows contributed to the unequal impact of COVID-19 across the state, even though these determinants were not formally included in our models.

The progression of epidemic waves contextualizes these patterns. The first wave, in mid-2020, was moderate yet sufficient to expose the limitations in the state’s hospital system and epidemiological surveillance. The most severe phase occurred between March and May 2021, when fatality rates reached their peak. The rapid expansion of the Gamma (P.1) variant, followed by the introduction of Delta, accounted for much of the severity observed during this period [13,23,35,45]. Alongside higher viral transmissibility, limited vaccine coverage and hospital overload further intensified the impact, with fatality rates surpassing 3% at certain points [37].

In early 2022, the emergence of the Omicron variant triggered a wave characterized by record infection numbers but proportionally low mortality. This phase reflects the protective effect of accumulated population immunity, derived from vaccination and prior exposures, which substantially mitigated severe outcomes even amid intense viral circulation, as also noted by Salzberger et al. (2021) and Gomes et al. (2024) [9,16,46].

Between 2023 and 2025, the epidemic curve exhibited only minor oscillations, indicating stable viral circulation sustained by localized transmission chains and very low lethality [38,47]. The contrast between the early crisis years and the final endemic stage underscores the combined effects of widespread immunization, expanded healthcare capacity, and the adaptation of preventive practices [39,47].

Previous surveillance data in Tocantins indicated that young adults (20–49 years) concentrated most infections, whereas mortality disproportionately affected the elderly [48]. This discrepancy reinforces the role of the economically active population as the main driver of transmission, while physiological vulnerability and comorbidities in older individuals contributed to severe outcomes [49]. At the pandemic’s peak in 2021, older adults accounted for the majority of deaths, supporting the prioritization of vaccination for elderly and immunosuppressed groups [50]. After 2022, the marked decline in mortality among older adults confirmed the protective effect of immunization, consistent with international evidence demonstrating substantial reductions in mortality following vaccination in these age groups [51].

State-level data also show that women were more frequently diagnosed (56% of cases), while men accounted for most deaths (59%) [52]. This suggests that, although women may have been more exposed through occupational and social factors, they sought testing and healthcare more frequently, resulting in higher detection rates [53]. Men, in turn, exhibited a higher prevalence of comorbidities and lower adherence to preventive measures and vaccination, potentially contributing to higher fatality [54]. These disparities were most pronounced in 2020–2021 and following the expansion of vaccination coverage [16,24].

Annual trends further corroborate these patterns: 2021 concentrated the greatest burden of morbidity and mortality, with cases and deaths more than doubling compared with the previous year [55]. From 2022 onward, despite more than 124,000 confirmed cases, mortality declined by over 90% [56]. This pattern persisted in subsequent years, with residual numbers in 2023 and 2025; only 2024 exhibited a slight increase, possibly linked to the circulation of Omicron subvariants [10].

Taken together, these findings show that the pandemic affected population groups in Tocantins unevenly: young adults were more exposed, older adults were more vulnerable to death, and men faced a greater risk of severe outcomes [57]. From 2022 onward, sustained immunization and health-system adaptation consolidated a low-severity endemic pattern [58]. These patterns underscore the need for continued protection of older adults and men, as well as surveillance capable of anticipating outbreaks in vulnerable subgroups [59].

In Tocantins, these unequal impacts are quantitatively reflected in the age- and sex-stratified patterns documented in our results (Figure S1A,B). Working-age adults (20–49 years) accounted for most infections, whereas mortality increased sharply from age 60 upward and was highest among those ≥70 years, confirming the disproportionate vulnerability of older adults. At the same time, women accounted for most diagnosed cases, whereas men concentrated the majority of deaths, suggesting gendered differences in exposure, health-seeking behavior, and the prevalence of comorbidities. These gradients likely underestimate the true burden in remote and socioeconomically vulnerable areas: municipalities with limited diagnostic capacity, longer travel times to referral hospitals, and weaker surveillance systems are more prone to underreport mild infections and under-ascertain COVID-19 deaths [60,61]. Thus, the observed inequalities almost certainly represent conservative estimates of the pandemic’s impact on vulnerable groups.

Vaccination represented the most decisive turning point in Tocantins, consistently altering the trajectory all major epidemiological indicators [62]. In 2021, the rapid expansion of coverage coincided with a progressive decline in mortality, from a weekly average of 77 deaths at the start of the campaign to only 3 following widespread vaccination. Hospitalizations followed the same pattern, falling from 133 to 7.3 weekly admissions, confirming that immunization primarily mitigated severe outcomes [16,17]. Even during the Omicron-driven surge in 2022, mortality remained low, highlighting the effectiveness of vaccination in decoupling incidence from lethality, as also reported by Lamarca et al. (2023) and Souza et al. (2025) [10,12].

Statistical analyses further strengthen this interpretation. ANOVA and Tukey post hoc tests showed significant differences between across pre-vaccination, initial campaign, and widespread vaccination periods for all indicators (*p* < 0.001) [63]. GAMs revealed clear dose–response associations, with the strongest explanatory power for hospitalizations (adjusted R^2^ = 0.907) and deaths (0.887) [64]. ITS models documented marked reversals in growth trends following vaccination, indicating sustained post-intervention declines even without abrupt level changes [65]. Seasonal decomposition (STL) corroborated a continuous downward trend from 2022 onward [66].

Together, these analyses demonstrate that vaccination not only reduced COVID-19 severity but also reshaped the epidemiological regime of the state, enabling a transition to low transmission and low severity from 2023 onward [67]. Consistent with findings elsewhere, the data from Tocantins show that increases in vaccination coverage were accompanied by approximately 90% declines in hospital pressure and mortality [68]. These reductions likely reflect the combined effects of vaccination, infection-acquired immunity (“hybrid immunity”), improvements in clinical care, and enhanced health-system capacity. Overall, the experience of Tocantins suggests that vaccination—together with accumulated natural immunity and other public health measures—was the key driver associated with COVID-19 control in the state, as consistently indicated by multiple time-series approaches [69]. These results highlight the importance of maintaining booster strategies and continuous surveillance to contain localized outbreaks and anticipate the emergence of new variants [70].

Genomic surveillance carried out in Tocantins between September 2020 and May 2025 reveals a diverse and continually evolving scenario of SARS-CoV-2 circulation. Sequencing of nearly 4000 genomes enabled the identification of both the broad lineage diversity and the chronology of viral replacements that accompanied the major phases of the pandemic [17]. The spatial distribution shows a strong concentration of sequences in Palmas, responsible for more than half of all genomes, reflecting its higher population density and centralized health infrastructure. In contrast, smaller or remote municipalities were underrepresented, highlighting logistical limitations in sample collection and transport, a pattern also documented elsewhere in northern Brazil [25]. This sampling inequality reinforces the need to decentralize genomic surveillance in order to improve regional representativeness.

From an evolutionary perspective, the state’s trajectory broadly mirrored global patterns. B.1.1 and P.2 lineages dominated circulation in 2020, followed by the rapid rise of Gamma (P.1) in 2021 and subsequent replacement by Delta later that year [23,34]. In 2022, the introduction of Omicron triggered the largest wave of infections, accompanied by rapid turnover among its sublineages (BA.1, BA.2, BA.4, BA.5), illustrating the high adaptive capacity of this variant group [10,12]. In the following years, a sequential pattern of within-Omicron replacements was observed: in 2023, BQ.1/BQ.1.1 and BE circulated initially, later supplanted by XBB, JD, and JN, which predominated into early 2024. In 2025, a new shift occurred with the rise of LP.8.1.4 and the emergence of PD sublineages, while other variants, such as KP and residual JN lineages, persisted at low frequency.

By 2025, SARS-CoV-2 circulation in Tocantins was dominated by Omicron sublineages of the LP.8.1 family, particularly LP.8.1.4. LP.8.1 is a JN.1 descendant that evolved from KP.1 and carries five additional spike mutations beyond KP.3.1.1—F186L and R190S in the N-terminal domain, R346T and H445R in the receptor-binding domain, and K1086R in S2—while its sublineage LP.8.1.1 contains an additional K679R substitution near the furin cleavage site [71]. This constellation of mutations modifies key antigenic and receptor-binding residues but does not substantially increase neutralization escape relative to other recent JN.1 derivatives such as KP.3.1.1 and XEC [71,72]. Experimental data indicate that LP.8.1 has a modest effective reproduction number advantage over contemporaneous XEC in some settings and shows lower pseudovirus infectivity but greater immune resistance than JN.1, with a comparable degree of neutralization escape to other JN.1 descendants [72]. Importantly, current evidence does not suggest increased intrinsic severity for LP.8.1 relative to other Omicron subvariants [71,72]. In Tocantins, the expansion of LP.8.1.4 occurred against a backdrop of high vaccination coverage, stable low mortality, and residual hospitalization rates, supporting the interpretation that its success reflects enhanced transmission fitness in an immunized population rather than any increase in virulence. This pattern exemplifies the late-pandemic transition to an endemic phase dominated by successive Omicron descendants that optimize transmission under strong immune pressure while severe outcomes remain largely mitigated by vaccine- and infection-derived immunity.

Similar late-pandemic dynamics, characterized by successive variant replacements occurring on a background of rising immunity and progressively attenuated severe outcomes, have been interpreted within kinetic and thermodynamic epidemic frameworks as the result of variant competition across an evolving fitness landscape shaped by interventions, behavioral responses, and host immunity [42,43,44]. From this perspective, the orderly succession from Gamma and Delta to early Omicron lineages and, more recently, to JN- and LP.8.1-related subvariants in Tocantins reflects shifts in effective transmission “rates” and control “reaction” strengths, rather than abrupt regime changes, providing a mechanistic complement to the descriptive patterns documented here.

The detection of 166 lineages—more than 30% of which were identified only once—highlights Tocantins as both a site of sporadic introductions and sustained transmission. Regionally, Tocantins occupies a strategic position linking Brazil’s North and Midwest macroregions through major transportation routes, particularly the BR-153 highway [20,21,23]. The lineage turnover documented here largely mirrored patterns in neighboring states, with repeated detections of lineages first identified or predominant elsewhere in Brazil and little evidence of local lineage emergence [73]. This suggests that Tocantins may have functioned primarily as a transit node that received and redistributed variants along regional mobility axes, rather than as a major source of novel viral diversity [20,21,23]. In this descriptive sense, the state can be viewed as a potential “variant corridor” between the North and Midwest; however, this interpretation remains inferential and should be validated through future multi-state phylogeographic analyses employing harmonized sampling and explicit models of viral flow.

The recent predominance of LP.8.1.4 and PD lineages in 2025 underscores the need to maintain active genomic surveillance, given their potential to sustain local transmission chains. Moreover, the spatial and temporal heterogeneity in sequencing intensity indicates that expanding and decentralizing genomic surveillance is essential for anticipating risk scenarios and guiding public health decision-making [74]. Integrating these genomic findings with epidemiological data illustrates how variant succession aligned closely with the severity and volume of cases observed during pandemic waves [75]. Together, these results demonstrate that integrated genomic and epidemiological surveillance can support timely, regionally tailored responses to emerging variants.

This study has limitations that should be considered when interpreting the findings. First, reliance on officially reported cases and deaths likely underestimates true incidence, particularly in remote areas with limited testing capacity. Second, genomic surveillance, although extensive, was unevenly distributed across municipalities, potentially biasing lineage representation. Third, we did not quantify the influence of sociodemographic or mobility factors: municipal-level indicators of income, occupation, social vulnerability, or intermunicipal mobility were not incorporated into our time-series or regression models, limiting our ability to assess their contribution to regional transmission differences. Fourth, our time-series models did not explicitly parameterize time-varying vaccine effectiveness, waning immunity, or booster-specific effects; weekly vaccine doses and overall coverage served as ecological proxies, meaning that the associations reflect net population impact rather than individual-level protection dynamics. Despite these limitations, integrating epidemiological and genomic data provides a robust account of the pandemic’s trajectory in Tocantins and offers insights relevant not only for Brazil but also for other regions facing similar structural challenges.

## 5. Conclusions

This study shows that Tocantins experienced a clear transition from a high-burden pandemic phase in 2020–2021 to a stable endemic scenario characterized by low mortality from 2023 onward. Vaccination represented the decisive turning point, accompanying reductions of more than 90% in hospitalizations and deaths and driving a sustained decoupling between incidence and severity. In parallel, genomic surveillance documented sequential waves of variant replacement, culminating in the predominance of LP.8.1.4 in 2025 and illustrating the continued adaptive evolution of SARS-CoV-2. Together, these findings highlight two complementary lessons. First, immunization remains the most effective strategy to mitigate morbidity and mortality, even in the face of ongoing viral evolution. Second, continuous and decentralized genomic surveillance is essential for anticipating emerging threats and ensuring timely and equitable public health responses.

## Figures and Tables

**Table 1:** Results of one-way ANOVA and Tukey’s HSD post hoc tests for confirmed COVID-19 cases, deaths, hospitalizations, incidence, mortality, vaccination coverage, and the vaccine-to-case ratio across three vaccination periods in Tocantins, Brazil. Vaccination periods were defined as: pre-vaccination (up to week 2 of 2021), initial campaign (weeks 3–26 of 2021), and widespread vaccination (week 27 of 2021 onward). Abbreviations: PV = Pre-vaccination; IC = Initial campaign; WV = Widespread vaccination.

Outcome	df (Model/ Residual)	MS (Model/ Residual)	*F*-Value	*p*-Value	Significant Comparisons (Tukey HSD, 95% CI)
Confirmed Cases	2/290	1.32 × 10^8^/3.73 × 10^6^	35.5	<0.001	IC > PV (*p* < 0.001); WV > PV (*p* = 0.0106); WV > IC (*p* < 0.001)
Deaths	2/290	7.71 × 10^4^/3.81 × 10^2^	202.0	<0.001	IC > PV (*p* < 0.001); WV > PV (*p* < 0.001); WV > IC (*p* < 0.001)
Hospitalizations	2/290	2.26 × 10^5^/8.97 × 10^2^	252.0	<0.001	IC > PV (*p* < 0.001); WV > PV (*p* < 0.001); WV > IC (*p* < 0.001)
Incidence	2/290	5.16 × 10^5^/1.46 × 10^4^	35.5	<0.001	IC > PV (*p* < 0.001); WV > PV (*p* = 0.0108); WV > IC (*p* < 0.001)
Mortality	2/290	3.01 × 10^2^/1.49	202.1	<0.001	IC > PV (*p* < 0.001); WV > PV (*p* < 0.001); WV > IC (*p* < 0.001)
Vaccination Coverage	2/290	7.59 × 10^5^/4.85 × 10^2^	1564.0	<0.001	IC > PV (*p* < 0.001); WV > PV (*p* < 0.001); WV > IC (*p* < 0.001)
Vaccine-to-Case Ratio	2/290	9.71 × 10^4^/1.20 × 10^3^	8.11	<0.001	WV > PV (*p* = 0.0005)

**Table 2:** Generalized additive model (GAM) results assessing the association between weekly COVID-19 vaccination and key epidemiological indicators in Tocantins, Brazil, 2020–2025.

Outcome Variable	Adj. *R*^2^	Deviance Explained (%)	EDF (Vaccination)	*F*-Statistic	*p*-Value
Confirmed cases	0.796	81.1	4.66	35.13	***
Deaths	0.887	89.5	4.77	47.69	***
Hospitalizations	0.907	91.4	5.09	51.11	***
Incidence	0.615	64.0	4.23	16.17	***
Mortality	0.822	83.4	4.63	30.83	***
Vaccination Coverage	1	100	3.08	4.92	***
Vaccine-to-Case Ratio	0.524	55.6	3.69	6.38	***

EDF = Estimated degrees of freedom for the smooth term of weekly vaccine doses (7-week moving average). All models include additional smooth terms for time (in weeks) and seasonal variation. Significance levels: *p* < 0.001 ***.

**Table 3:** Results of Interrupted Time Series (ITS) analysis for key COVID-19 indicators in Tocantins, Brazil. Outcomes were modeled using segmented linear regression to evaluate changes associated with the start of the vaccination campaign (intervention point: epidemiological week 3 of 2021). β_1_ represents the pre-intervention trend (weekly change), β_2_ the immediate level change after the intervention, and β_3_ the post-intervention changes in slope. Significant negative β_3_ values (*p*  <  0.001) across all indicators indicate sustained reductions in transmission and severity following vaccination. Adjusted R^2^ values denote overall model fit.

Outcome	β_1_	*p*-Value β_1_	β_2_	*p*-Value β_2_	β_3_	*p*-Value β_3_	Adj. *R*^2^
Confirmed cases	+52.44	<0.001	+64.45	0.881	−70.14	<0.001	0.395
Deaths	+0.62	<0.001	−0.81	0.919	−0.88	<0.001	0.345
Hospitalizations	+0.99	<0.001	+2.35	0.853	−1.46	<0.001	0.423
Incidence rate	+3.40	<0.001	+0.51	0.988	−4.49	<0.001	0.284
Mortality rate	+0.04	<0.001	−0.08	0.889	−0.06	<0.001	0.306

β_1_ = slope before intervention; β_2_ = immediate level change after vaccination campaign start (week 3 of 2021); β_3_ = change in trend post-intervention.

## Data Availability

The authors declare that all data supporting the findings of this study are available within the paper. The SARS-CoV-2 genomes were obtained from the GISAID database and were described in Table S2.

## Supplementary Materials

The following supporting information can be downloaded at: https://www.mdpi.com/article/10.3390/v17111521/s1. Figure S1. Age-stratified distribution of COVID-19 cases and deaths in Tocantins (2020–2025). Figure S2. Distribution of key COVID-19 epidemiological indicators across vaccination phases in Tocantins, Brazil (2020–2025). Figure S3. Partial effect plots of weekly COVID-19 vaccination on key epidemiological indicators in Tocantins, Brazil. Figure S4. Seasonal–trend decomposition using Loess (STL) applied to weekly COVID-19 epidemiological time series in Tocantins, Brazil (January 2020–July 2025). Figure S5. Spatial distribution of SARS-CoV-2 genome sequences obtained in Tocantins, Brazil, from September 2020 to May 2025. Figure S6. SARS-CoV-2 lineages detected in Tocantins, Brazil, from September 2020 to May 2025. Figure S7. Emerging SARS-CoV-2 lineages in Tocantins visualized with Nextstrain/Auspice. Table S1. SARS-CoV-2 genomic surveillance in Tocantins by municipality (study period). Table S2. Data availability (GISAID Identifier: EPI_SET_251017vr).
