# Supplementary material for: Five Years of COVID-19 in Tocantins, Brazil: Epidemiology, Vaccination Impact, and SARS-CoV-2 Genomic Dynamics (2020–2025)
Source: Viruses. 2025 Nov 20;17(11):1521. doi: 10.3390/v17111521 (PMC12656998; doi:10.3390/v17111521)
Supplement: Supplementary file 1 [file viruses-17-01521-s001.zip › viruses-3966484-Supplementary.pdf]

*Supplementary Materials*

# Five Years of COVID-19 in Tocantins, Brazil: Epidemiology, Vaccination Impact, and SARS-CoV-2 Genomic Dynamics (2020 - 2025)

**Olivia de Souza da Conceição[1†], Ueric José Borges de Souza[1*†], Franciano Dias Pereira Cardoso[2], Evgeni Evgeniev Gabev[3], Bergmann Morais Ribeiro[4], Gil Rodrigues dos Santos[5], Renisson Neponuceno de Araújo Filho[6], Marcos Gontijo da Silva[7], Fernando Rosado Spilki[8], and Fabrício Souza Campos[1,9*]**

1 - Bioinformatics and Biotechnology Laboratory, Campus of Gurupi, Federal University of Tocantins, Gurupi 77410-570, TO, Brazil
2 - Central Public Health Laboratory of the State of Tocantins, Palmas 77054-970, Brazil
3 - Department of Physiology and Pathophysiology, Medical University of Sofia, Sofia 1431, Bulgaria
4 - Baculovirus Laboratory, Department of Cell Biology, Institute of Biological Sciences, University of Brasilia, Brasília 70910-900, DF, Brazil
5 - Laboratory of Phytopathology, Federal University of Tocantins, Gurupi 77402-970, TO, Brazil
6 - Universidade Federal Rural de Pernambuco, Departamento de Tecnologia Rural, Recife 52171-900, PE, Brazil. E-mail: renisson.neponuceno@ufrpe.br, Orcid: 0000-0002-9747-1276
7 - Programa de Pós-graduação em Biotecnologia da Universidade Federal do Tocantins (UFT). Gurupi 77402-970, TO, Brazil. E-mail: gontijobio@uft.edu.br, Orcid: 0000-0002-6474-6640
8 - Molecular Microbiology Laboratory, Feevale University, Novo Hamburgo 93525-075, RS, Brazil
9 - Laboratório de Bioinformática & Biotecnologia, Instituto de Ciências Básicas da Saúde, Universidade Federal do Rio Grande do Sul, Porto Alegre 90010-150, RS, Brazil

*Authors to whom correspondence should be addressed.
† These authors contributed equally to this work.
* Correspondence: uericjose@gmail.com; camposvet@gmail.com

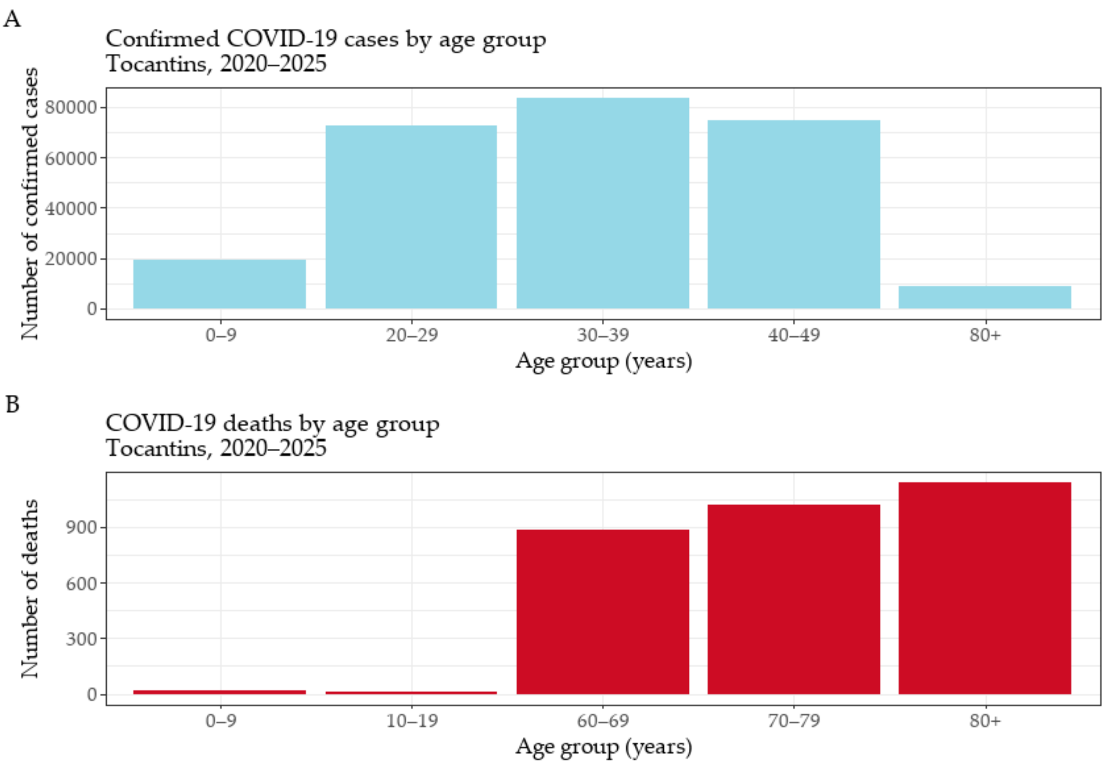


**Figure S1.** Age-Stratified Distribution of COVID-19 Cases and Deaths in Tocantins (2020–2025). A. Absolute number of confirmed COVID-19 cases by 10-year age groups in Tocantins (2020–2025). Bars show the predominance of infections among adults aged 20–49 years, with a peak in the 30–39-year group. B. Absolute number of COVID-19 deaths by 10-year age groups in Tocantins (2020 - 2025). Mortality increases sharply with age and is concentrated among individuals aged ⩾70 years.

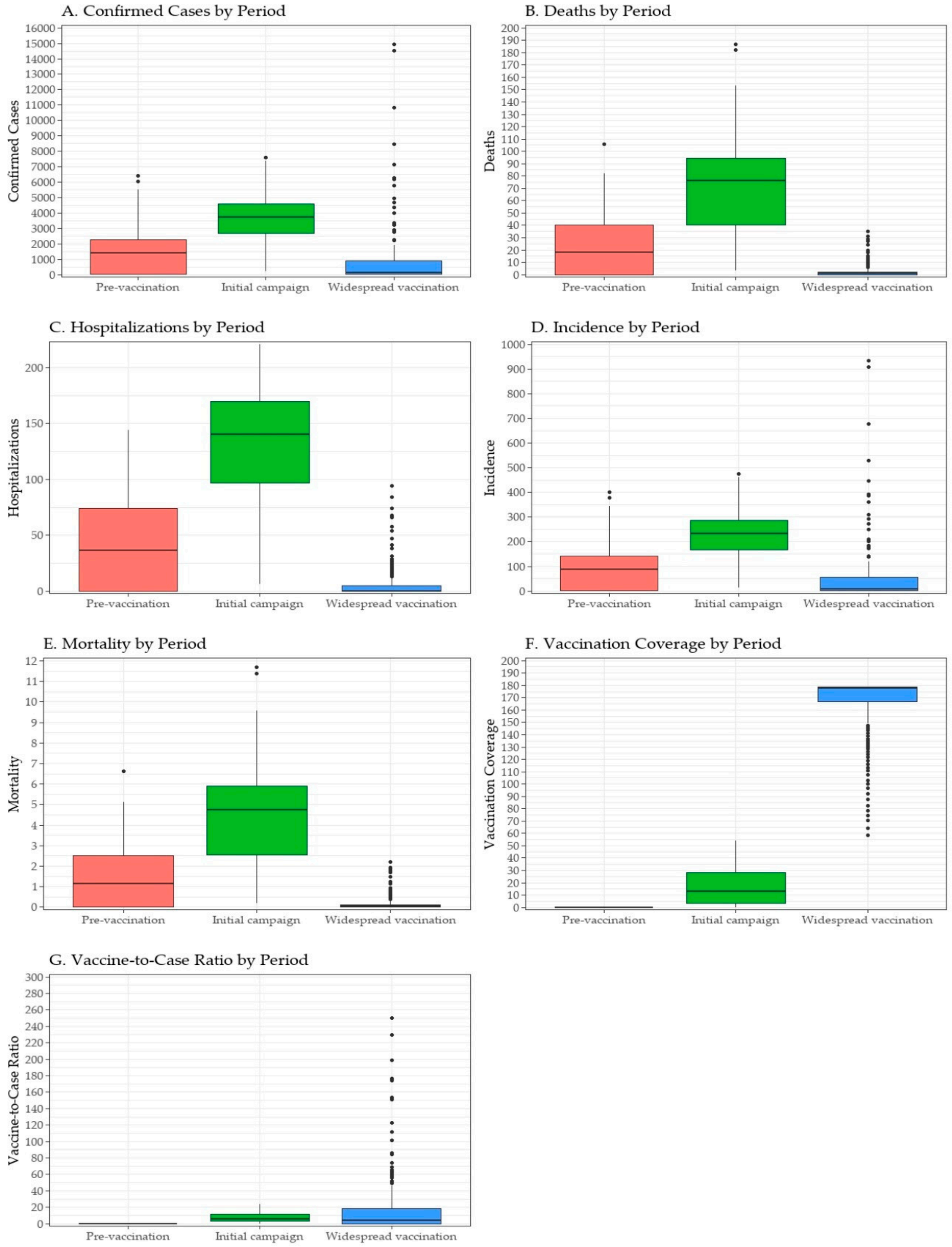


**Figure S2.** Distribution of key COVID-19 epidemiological indicators across vaccination phases in Tocantins, Brazil (2020–2025). Boxplots show weekly values for: (A) confirmed cases, (B) deaths, (C) hospitalizations, (D) incidence per 100,000 inhabitants, (E) mortality rate per 100,000 inhabitants, (F) vaccination coverage (%), and (G) vaccine-to-case ratio, stratified across three epidemiological periods: pre-vaccination, initial campaign, and widespread vaccination. Boxes represent the interquartile range (IQR), with horizontal lines indicating medians; whiskers extend to 1.5 × IQR, and points denote outliers. ANOVA results showed significant differences across all periods for all indicators ($p < 0.001$).

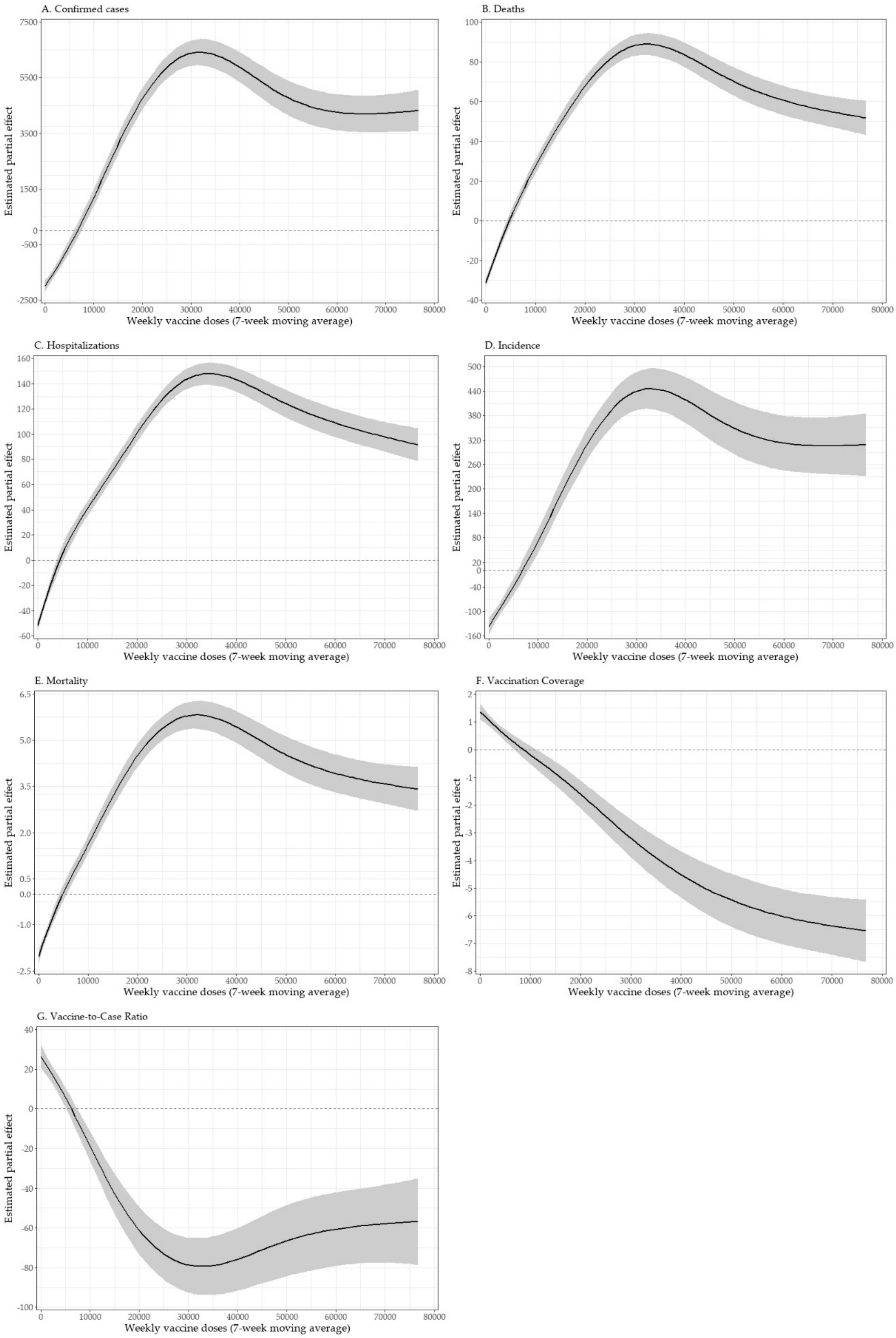


**Figure S3**. Partial-effect plots showing the association between weekly COVID-19 vaccination and key epidemiological indicators in Tocantins, Brazil: (A) confirmed cases, (B) deaths, (C) hospitalizations, (D) incidence, (E) mortality, (F) vaccination coverage, and (G) vaccine-to-case

ratio. Shaded areas represent 95% confidence intervals. All models were adjusted for long-term temporal trends (weeks) and seasonal variation (ISO week).

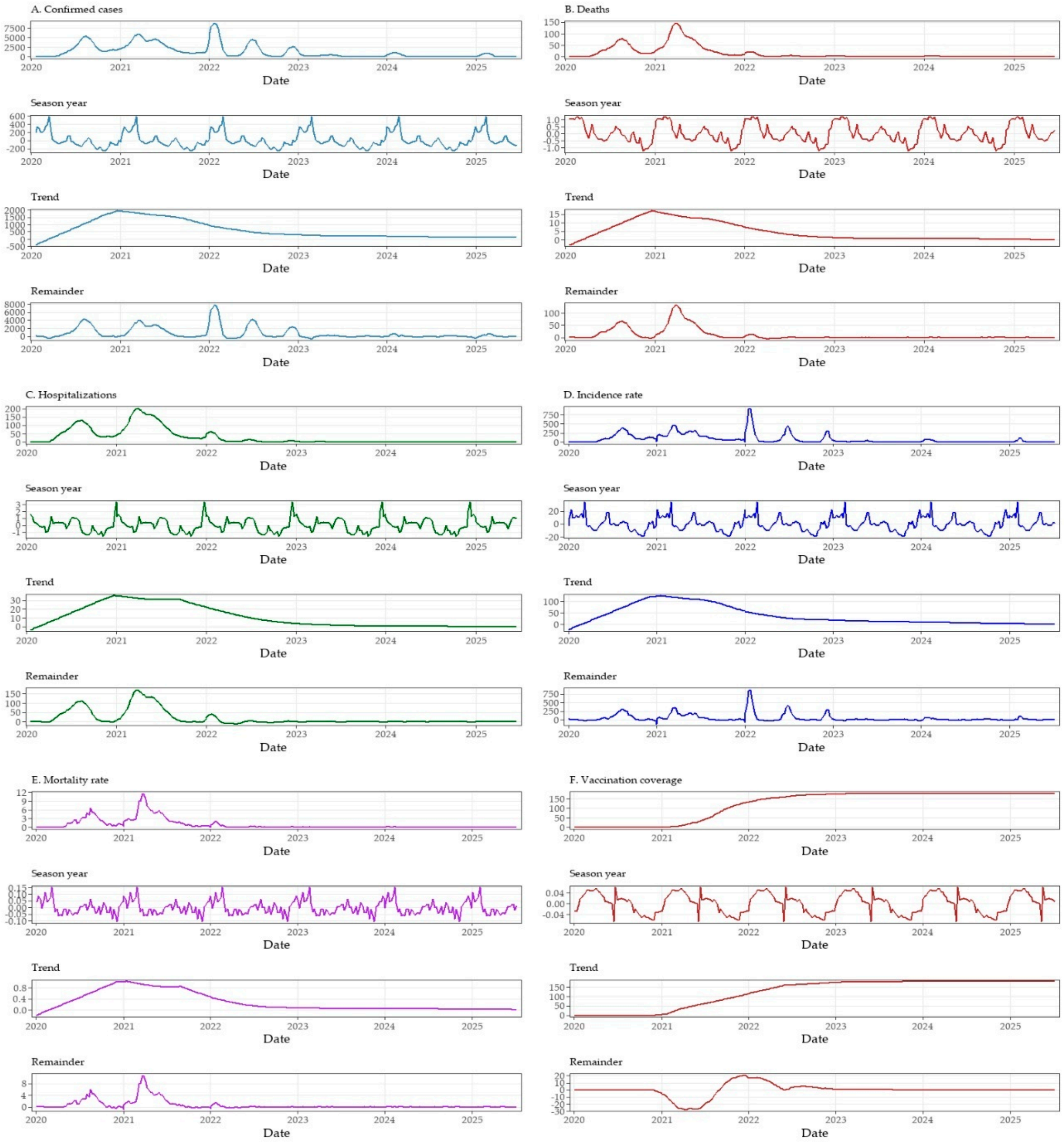


**Figure S4.** Seasonal-Trend Decomposition using Loess (STL) applied to weekly COVID-19 epidemiological time series in Tocantins, Brazil (January 2020–July 2025). Each panel (A–F) shows the decomposition of a given indicator into four components: Observed (original series), Seasonal (recurring intra-annual patterns), Trend (long-term trajectory), and Remainder (irregular fluctuations). Indicators include: (A) confirmed cases, (B) deaths, (C) hospitalizations, (D) incidence rate per 100,000 inhabitants, (E) mortality rate per 100,000 inhabitants, and (F) vaccination coverage. Pronounced seasonal patterns with recurrent mid-year peaks were evident for confirmed cases, incidence, and deaths, whereas trend components captured the progression of epidemic waves and the effects of vaccination.

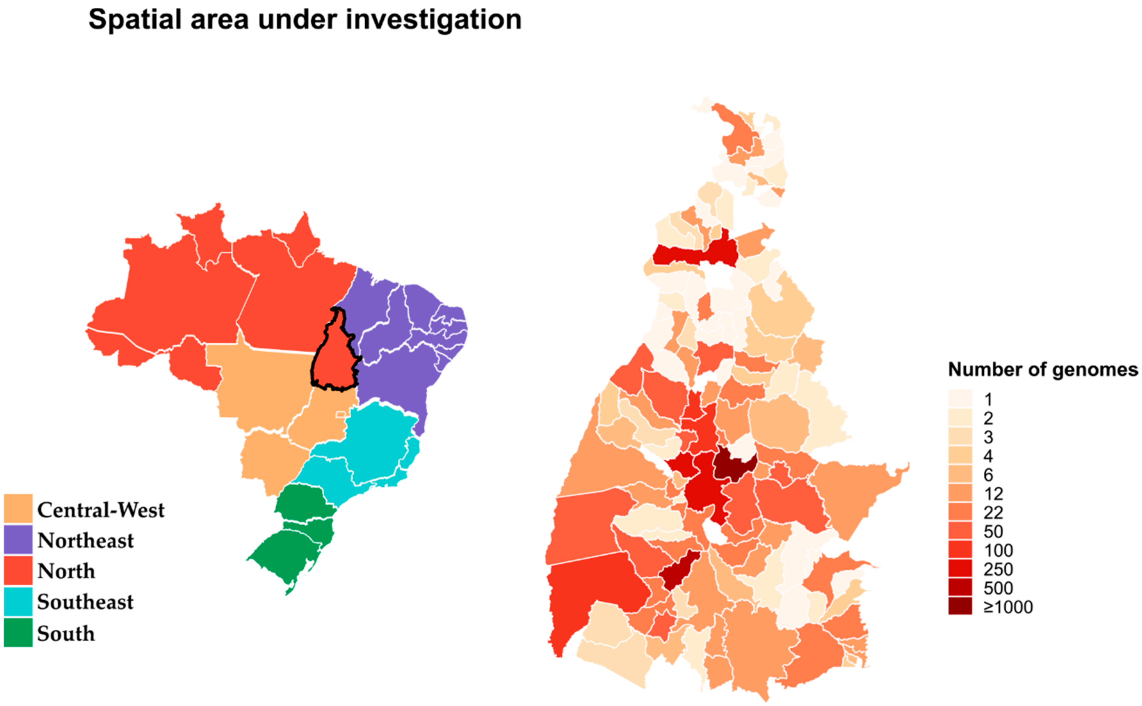


**Figure S5**. Spatial distribution of sequenced SARS-CoV-2 genomes in Tocantins, Brazil, from September 2020 to May 2025. Left: Political map of Brazil highlighting its geographic regions, with the state of Tocantins outlined in black. Right: Choropleth map of Tocantins showing the number of genomes sequenced per municipality. Color intensity reflects sequencing volume, with the highest counts observed in Palmas and surrounding municipalities. Genomic data were obtained from the GISAID EpiCoV database, and cartographic layers (municipal and state boundaries) were sourced from the IBGE digital cartographic database using the geobr package in R.

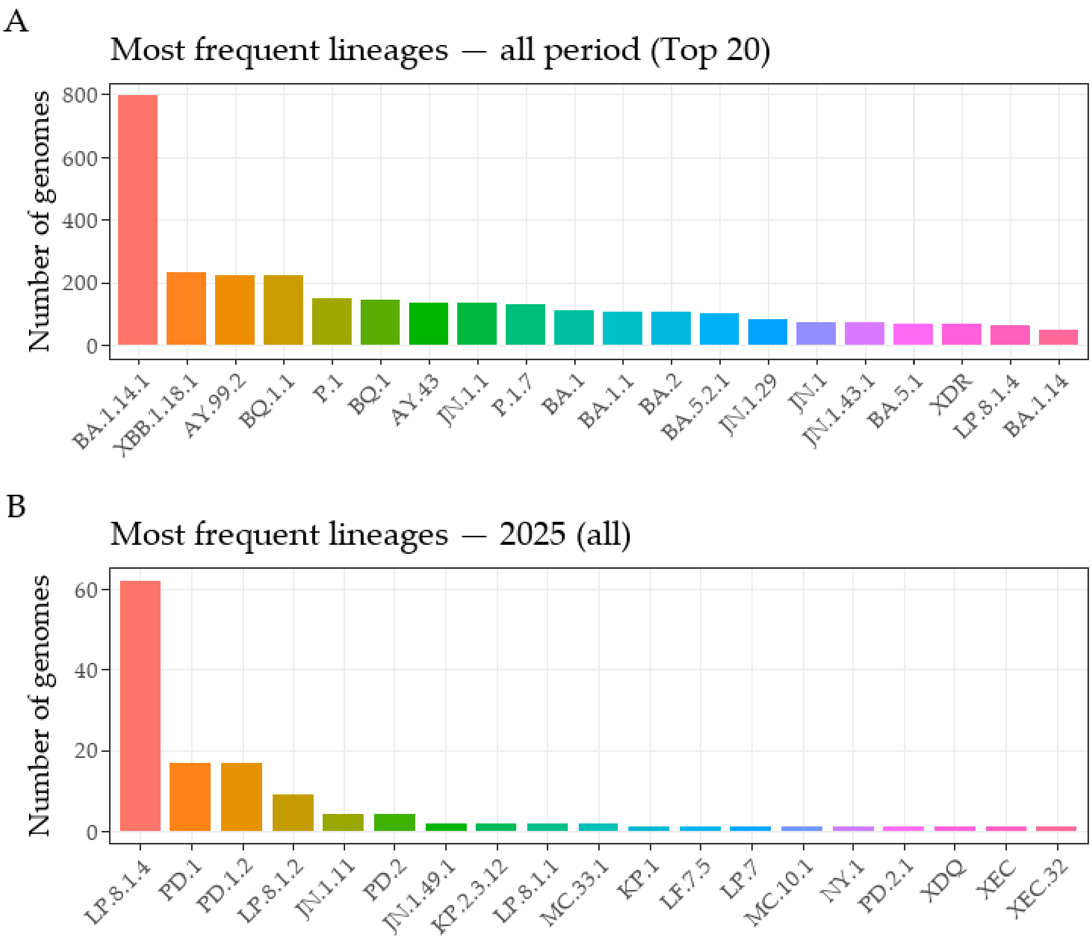


**Figure S6**. SARS-CoV-2 lineages identified in Tocantins, Brazil, from September 2020 to May 2025. (A) Top 20 most frequently detected lineages across the entire study period, showing the predominance of BA.1.14.1, XBB.1.18.1, AY.99.2, BQ.1.1, and P.1. (B) Lineage distribution from January to May 2025, highlighting the dominance of LP.8.1.4 and the emergence of PD sublineages (PD.1, PD.1.2, PD.2), along with sporadic detections of additional variants. Counts are based on SARS-CoV-2 genomes obtained from GISAID, with monthly relative frequencies normalized to 100%.

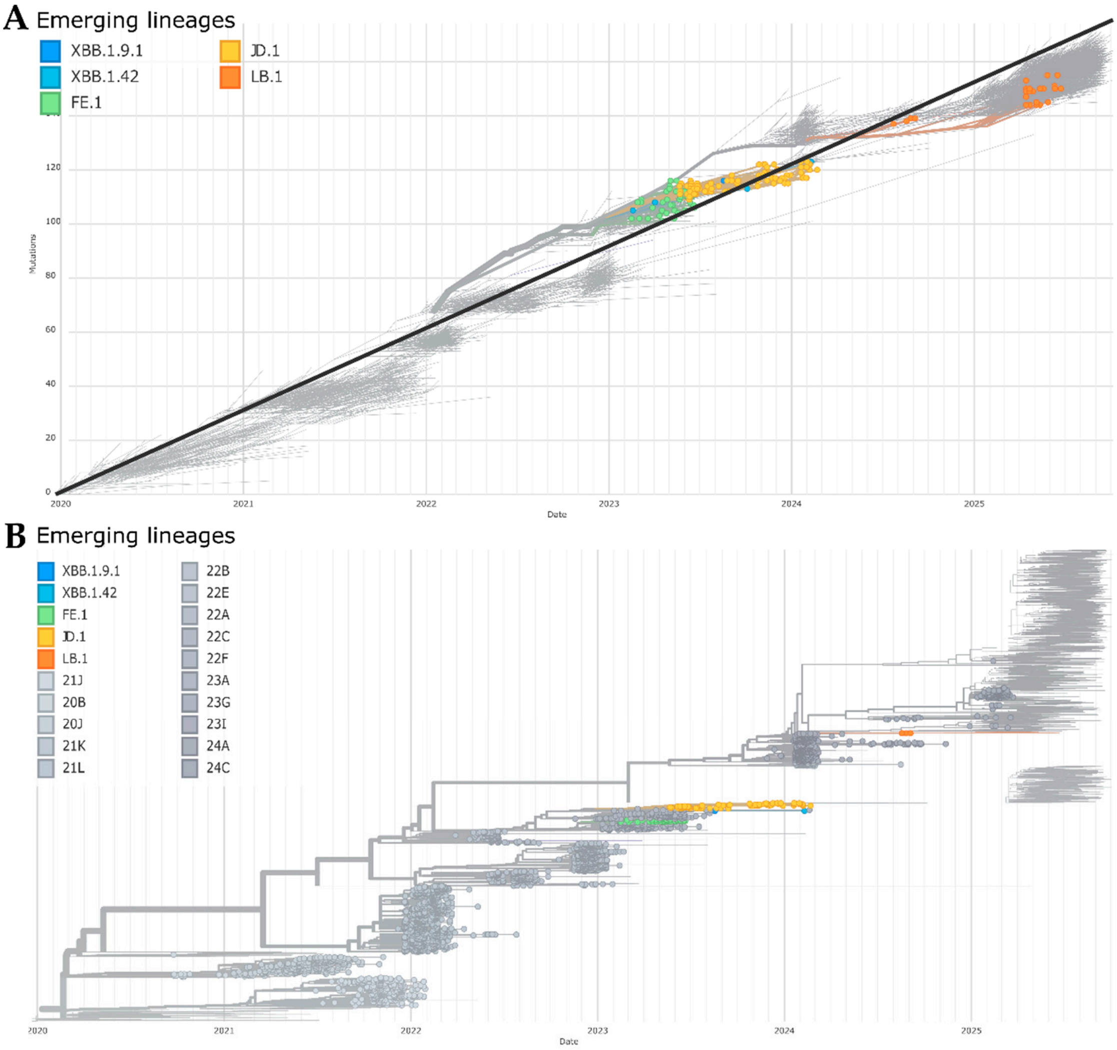


**Figure S7.** Emerging SARS-CoV-2 lineages in Tocantins, Brazil, as identified through the Nextstrain/Auspice workflow. (A) Root-to-tip regression plot highlighting emerging lineages detected in Tocantins—XBB.1.9.1, XBB.1.42, FE.1, JD.1, and LB.1 (colored points)—positioned against the global background dataset (gray). (B) Time-scaled phylogeny showing the same emerging lineages embedded within the global tree (colored tips), with all other sequences displayed in gray. The highlighted clades illustrate the recent expansion and phylogenetic placement of these lineages within international SARS-CoV-2 diversity.

**Table S1.** SARS-CoV-2 genomic surveillance in Tocantins, Brazil, by municipality (September 2020–May 2025). For each municipality, the table reports the number of distinct Pango lineages detected and the corresponding lineage identities. Lineage designations follow the Pango nomenclature system current at the time of analysis.

| Location | Number of genomes | Number of linhagens | Pango Lineages |
|---|---|---|---|
| Palmas | 1895 | 132 | AY.101, AY.116, AY.34, AY.34.1.1, AY.42, AY.43, AY.43.2, AY.44, AY.46.3, AY.99.1, AY.99.2, B.1.1, B.1.1.28, B.1.617.2, BA.1, BA.1.1, BA.1.1.1, BA.1.1.14, BA.1.1.18, BA.1.14, BA.1.14.1, BA.1.14.2, BA.1.15, BA.1.17, BA.1.17.2, BA.1.18, BA.1.9, BA.2, BA.2.12.1, BA.2.56, BA.2.81, BA.2.9, BA.4, BA.4.1, BA.4.6, BA.5, BA.5.1, BA.5.1.15, BA.5.1.22, BA.5.2.1, BE.10, BE.9, BQ.1, BQ.1.1, BQ.1.1.18, BQ.1.1.36, BQ.1.1.5, BQ.1.1.60, BQ.1.18, BQ.1.5, CK.1.4, CV.1, DL.1, EF.1.1.1, EG.1, EG.5.1.1, EY.1, FE.1, FE.1.1.1, FE.1.2, GK.1, GK.1.1, JD.1, JD.1.1, JD.1.1.1, JD.1.2, JD.2, JN.1, JN.1.1, JN.1.1.3, JN.1.1.5, JN.1.11, JN.1.14, JN.1.16, JN.1.16.1, JN.1.16.3, JN.1.18, JN.1.20, JN.1.23, JN.1.29, JN.1.29.1, JN.1.3, JN.1.30, JN.1.4, JN.1.4.5, JN.1.43, JN.1.43.1, JN.1.49.1, JN.1.51, JN.1.55.2, JN.1.61, JN.1.7, JN.1.8.1, JN.2, JN.2.5, KP.2.18, KP.2.3.12, KR.5, LB.1, LB.1.4, LB.1.7, LF.7.5, LP.8.1.1, LP.8.1.2, LP.8.1.4, MC.10.1, MC.13, MC.33.1, MJ.1, MJ.2, NG.1, NY.1, P.1, P.1.7, P.2, PD.1, PD.1.2, PD.2, PD.2.1, XBB, XBB.1, XBB.1.16, XBB.1.18, XBB.1.18.1, XBB.1.5, XBB.1.5.19, XBB.1.5.59, XBB.1.5.7, XBB.1.5.70, XBB.1.5.86, XDR, XEC |
| Gurupi | 262 | 57 | AY.43, AY.99.2, B.1.1.28, BA.1, BA.1.1, BA.1.1.1, BA.1.14, BA.1.14.1, BA.2, BA.2.12.1, BA.2.3, BA.2.81, BA.2.9, BA.4, BA.4.6, BA.5.1, BA.5.2.1, BA.5.3.1, BE.9, BQ.1, BQ.1.1, BQ.1.13, DL.1, EG.1, FE.1.1, FE.1.2, JD.1, JD.1.1, JD.1.1.1, JN.1, JN.1.1, JN.1.23, JN.1.29, JN.1.29.1, JN.1.43, JN.1.43.1, KP.2.3, KP.3.1.1, KR.5, LP.7, LP.8.1.4, MC.11.3, MC.27, MJ.1, MJ.1.1, P.1, P.1.14, P.1.7, P.2, PD.1, XBB, XBB.1.18, XBB.1.18.1, XBB.1.5, XBB.1.5.70, XDR, XDR.1.1 |
| Porto Nacional | 210 | 39 | AY.124.1, AY.34, AY.43, AY.46.3, AY.99.2, B.1.617.2, BA.1, BA.1.1, BA.1.14, BA.1.14.1, BA.2, BA.2.48, BA.2.81, BA.4, BA.5.1, BE.10, BE.9, BQ.1, BQ.1.1, CK.1.4, DL.1, FE.1, JD.1.1.1, JN.1, JN.1.1, JN.1.29, JN.1.43.1, JN.1.61, KR.5, LP.8.1.2, LP.8.1.4, MJ.2, P.1, P.1.7, PD.1, PD.1.2, XBB.1.18.1, XBB.1.5, XDR |

| | | | |
|---|---|---|---|
| Araguaína | 163 | 38 | AY.43, AY.99.2, B.1.1, B.1.1.28, BA.1, BA.1.1, BA.1.14, BA.1.14.1, BA.1.17.2, BA.2, BA.5.1, BA.5.2.1, BE.10, BF.41.1, BQ.1, BQ.1.1, DL.1, GK.1, HV.1, JD.1.1, JN.1, JN.1.1, JN.1.11, JN.1.29, JN.1.43.1, KP.2.3.12, KR.5, LP.8.1.4, P.1, P.1.7, P.2, PD.1.2, PD.2, XBB.1.18, XBB.1.5, XBB.1.5.70, XDQ, XDR |
| Paraíso do Tocantins | 113 | 33 | AY.99.2, BA.1, BA.1.1, BA.1.1.14, BA.1.14, BA.1.14.1, BA.1.20, BA.5.1, BA.5.2.1, BQ.1.1, FE.1.1.4, FE.1.2, JD.1, JD.1.1.1, JD.2, JN.1, JN.1.1, JN.1.29.1, JN.1.4, JN.1.43.1, JN.1.7, KP.1, LP.8.1.4, P.1, P.1.7, P.2, PD.1, XBB.1.18.1, XBB.1.5, XBB.1.5.59, XBB.1.5.70, XBB.1.5.86, XDR |
| Formoso do Araguaia | 73 | 26 | AY.99.2, B.1.1.28, BA.1, BA.1.1, BA.1.14.1, BA.1.17, BA.1.17.2, BA.2, BA.2.12.1, BA.2.81, BA.4, BA.5.1, BA.5.2.1, BQ.1, BQ.1.1, BQ.1.10, DL.1, JD.1.1, JD.1.1.1, JN.1, JN.1.1, JN.1.43.1, P.1, P.1.7, XBB.1.18.1, XDR |
| Miracema do Tocantins | 65 | 31 | AY.43, BA.1, BA.1.1, BA.1.14.1, BA.1.17.2, BA.5.1, BA.5.3.1, BE.9, BQ.1, BQ.1.1, BQ.1.1.60, FE.1, GK.1, JD.1.1, JN.1, JN.1.1, JN.1.1.5, JN.1.29, JN.1.43.1, KR.5, LP.8.1.2, P.1, P.1.2, P.1.7, PD.1, XBB.1.18, XBB.1.18.1, XBB.1.5, XBB.1.5.70, XBB.2.3, XDR |
| Miranorte | 53 | 14 | AY.116, AY.43, AY.99.2, BA.1, BA.1.1, BA.1.14, BA.1.14.1, BA.1.15, BA.1.17.2, BQ.1, JD.1.1, P.1, P.1.7, XBB.1.5.86 |
| Alvorada | 42 | 17 | AY.34.1.1, BA.1, BA.1.1, BA.1.14.1, BA.2, BE.9, BQ.1, BQ.1.1, JN.1, JN.1.1, JN.1.16, JN.1.4, JN.1.43.1, P.1, P.1.7, XBB.1.18.1, XDR |
| Dueré | 39 | 15 | BA.1.14.1, BA.1.17.2, BA.2, BA.2.81, BA.4.1, BA.5.1.35, BA.5.2.1, BQ.1, BQ.1.1, DL.1, JN.1.1, JN.1.29, PD.1.2, XBB, XBB.1.18.1 |
| Araguacema | 35 | 18 | BA.1.14, BA.1.14.1, BA.2, BA.2.9, BA.4, BA.5.1, BA.5.2.1, BQ.1.1, BQ.1.1.36, JN.1, JN.1.1, LP.8.1.1, LP.8.1.4, P.1, P.1.7, PD.1, PD.1.2, XBB.1.18.1 |
| Lagoa da Confusão | 34 | 14 | AY.42, AY.43, AY.99.2, BA.1, BA.4, BA.5, BA.5.1, BA.5.1.22, BA.5.2.1, BQ.1.1, LP.8.1.4, P.1, PD.1.2, XEC.32 |
| Ponte Alta do Tocantins | 31 | 11 | BA.1, BA.1.14.1, BQ.1, BQ.1.1, DL.1, FE.1.2, JN.1.1, JN.1.29, JN.1.43.1, KR.5, XBB.1.18.1 |
| Lagoa do Tocantins | 29 | 8 | BA.1, BA.1.1, BA.1.14, BA.1.14.1, BA.2, BA.5.1, BA.5.2.1, CK.1.4 |
| Barrolândia | 25 | 13 | AY.99.2, BA.2, BA.5.1, BA.5.2.1, BE.10, BQ.1, BQ.1.1, JN.1, JN.1.1, LP.8.1.4, XBB.1.18, XBB.1.18.1, XBB.1.5.70 |
| Guaraí | 24 | 14 | AY.99.2, B.1.1.28, BA.1.1, BA.1.14.1, BA.1.17.2, BQ.1.1, JD.1.1, JN.1, JN.1.1, JN.1.55.2, JN.1.61, LP.8.1.4, P.1, P.1.7 |

| | | | |
|---|---|---|---|
| Monte do Carmo | 23 | 5 | AY.43, BA.1.14.1, BQ.1, DL.1, FE.1.2 |
| Dois Irmãos do Tocantins | 21 | 11 | BA.1.14.1, BA.4.6, BQ.1, BQ.1.1, DL.1, JD.1.1.1, JN.1, JN.1.1, JN.1.29, JN.1.43.1, XDR |
| Pedro Afonso | 21 | 11 | AY.99.2, BQ.1.1, JN.1, JN.1.1, JN.1.11, JN.1.43.1, JN.2, P.1, P.1.7, PD.1, XDR |
| Aliança do Tocantins | 21 | 11 | BA.1, BA.1.14.1, BA.4.6, BA.5.1, BA.5.2.1, BQ.1, BQ.1.1.4, JD.1.1, JN.1.29, JN.1.29.1, KR.5 |
| Arraias | 20 | 11 | BA.1, BA.1.1, BA.1.14, BA.1.14.1, BA.5.1, BA.5.2.1, BE.9, BQ.1.1, CK.1.4, P.1, P.1.7 |
| Silvanópolis | 20 | 10 | AY.43, AY.99.2, BA.1, BA.1.1, BA.1.14.1, JN.1.1, JN.1.18, JN.1.20, JN.1.29, KR.5 |
| Novo Acordo | 19 | 13 | AY.99.2, BA.1.1, BA.1.14.1, BA.2, BA.2.48, BA.5.1, BA.5.3.1, BQ.1, BQ.1.1, JD.1.1, JN.1.29, P.1, XBB.1.18.1 |
| Taguatinga | 18 | 9 | AY.42, BA.1.1, BA.1.14.1, BA.1.15, BQ.1, BQ.1.1, JD.1, XBB, XBB.1.18.1 |
| Cariri Do Tocantins | 18 | 11 | AY.43, BA.1.1, BA.1.14.1, BA.1.17.2, BA.5.1.22, BE.10, BQ.1.1, JN.1, JN.1.1, JN.1.29, XDR |
| Figueirópolis | 17 | 7 | AY.43, B.1.1.33, BA.1.1, BA.1.14, BA.1.14.1, P.1, P.1.7 |
| Araguatins | 16 | 6 | AY.122, AY.43, B.1.1.28, BA.1.14.1, BQ.1.1, XBB.1.18.1 |
| Brejinho de Nazaré | 16 | 7 | AY.43, AY.99.2, BA.1.1, BA.1.14, BA.1.14.1, P.1, XBB.1.18.1 |
| Dianópolis | 15 | 10 | AY.99.2, BA.2, BE.9, BQ.1, BQ.1.1, BQ.1.1.56, BQ.1.18, CK.1.4, JN.1.29, XBB.1.18.1 |
| Nova Rosalândia | 15 | 2 | BA.1, BA.1.14.1 |
| Colinas do Tocantins | 14 | 8 | AY.124.1, AY.34.1.1, AY.43.2, AY.99.2, BA.1.14.1, JN.1.1, P.1, XDR |
| Lajeado | 13 | 6 | BA.1.14.1, BA.5.1.22, LP.8.1.4, PD.1, XBB.1.18.1, XBB.1.5.59 |
| Santa Maria do Tocantins | 13 | 5 | AY.99.2, BA.1.14.1, BA.5.1, P.1.7, XBB.1.18.1 |
| Santa Rosa do Tocantins | 13 | 7 | AY.99.2, BA.1.1, BA.1.14, BA.1.14.1, BA.2, BQ.1, BQ.1.1.4 |
| Fátima | 13 | 7 | AY.99.2, BA.1, BA.1.1, BA.1.14.1, BA.5.1, BA.5.2.1, P.1.7 |
| Araguaiana | 12 | 4 | B.1.1, B.1.1.28, P.1, P.2 |
| Cristalândia | 12 | 8 | BA.1, BA.1.14, BA.1.14.1, BQ.1.1, GK.1, JD.1, JN.1.1, JN.1.43.1 |
| Santa Tereza do Tocantins | 11 | 5 | AY.99.2, BA.1, BA.1.14, BA.1.14.1, P.1 |
| Peixe | 11 | 8 | BA.1.1, BA.1.14.1, BA.2.81, FE.1.2, JD.1.1, JN.1.43, XBB.1.18.1, XBB.1.5.70 |
| Mateiros | 10 | 5 | AY.6, B.1.1.33, BA.1.14.1, BA.4, P.1 |
| Paranã | 10 | 5 | AY.99.2, BA.1.14.1, BA.5.2.1, P.1, P.1.7 |
| Rio dos Bois | 10 | 5 | AY.99.2, BA.1.1, BA.1.14.1, P.1.7, XBB.1.18.1 |

| | | | |
|---|---|---|---|
| São Salvador do Tocantins | 9 | 4 | BA.1.14.1, BA.4, BA.5.1, JD.1.1 |
| Aguiarnópolis | 9 | 7 | AY.43, BE.10, BQ.1, BQ.1.1, JN.1.1, JN.1.29, XBB.1.18.1 |
| Pindorama do Tocantins | 9 | 4 | AY.99.2, BA.1, BA.1.14.1, P.1 |
| Tocantínia | 9 | 6 | AY.99.2, BA.1, BA.1.14.1, JN.1.55.2, P.1.7, XBB.1.18.1 |
| São Bento do Tocantins | 8 | 5 | AY.43, BA.1.14.1, BA.1.17, BQ.1, DL.1 |
| Lavandeira | 8 | 3 | BA.2, BA.5.1, P.1.7 |
| Pequizeiro | 8 | 3 | BA.1.1, BA.1.14.1, BA.5.1 |
| Abreulândia | 8 | 7 | AY.43, AY.99.2, BA.1, BA.1.14.1, BA.5.1, BQ.1.1, P.1 |
| Aurora do Tocantins | 8 | 4 | BA.1.14.1, BN.1.3.1, JD.1.1, P.1.7 |
| Colmeia | 7 | 3 | AY.99.2, BA.1, BA.1.14.1 |
| Babaçulândia | 7 | 3 | AY.43, AY.99.2, BA.1.14.1 |
| Aragominas | 7 | 4 | AY.43, BA.1.14.1, JN.1.1, JN.1.43.1 |
| Pium | 7 | 4 | AY.99.2, BA.1.14.1, BA.5.2.1, P.1 |
| Chapada da Natividade | 7 | 7 | AY.43, BA.1.1, BA.1.1.1, BA.1.14.1, BA.2, BA.5.2.1, JD.1.1 |
| Palmeirópolis | 7 | 3 | BA.1.14.1, P.1, P.1.7 |
| Pugmil | 6 | 4 | BQ.1, DL.1, JN.1.1.5, XDR |
| Miracema | 6 | 3 | AY.43, AY.99.2, P.1.7 |
| Combinado | 6 | 2 | MC.33.1, P.1.7 |
| Rio Sono | 6 | 5 | AY.99.2, BA.1.14.1, BQ.1.1, JD.1.1, P.1 |
| Talismã | 5 | 1 | BA.1.14.1 |
| São Valério da Natividade | 5 | 1 | BA.1.14.1 |
| Nazaré | 5 | 3 | AY.43, BA.1.14.1, BA.5 |
| Recursolândia | 5 | 3 | BA.1, BA.1.14.1, XDR |
| Marianópolis do Tocantins | 5 | 4 | BA.1.14.1, BQ.1, PD.1.2, XBB.1.18.1 |
| Ponte Alta do Bom Jesus | 4 | 2 | BA.1.14.1, BA.1.17.2 |
| Novo Alegre | 4 | 3 | BA.1.14.1, BA.5.1, P.1 |
| Pau D'Arco | 4 | 3 | AY.34.1.1, BA.1.14.1, BA.1.17.2 |

| | | | |
|---|---|---|---|
| Augustinópolis | 4 | 2 | AY.43, P.1 |
| Caseara | 4 | 2 | BA.1.14.1, BQ.1 |
| Carmolândia | 4 | 4 | BA.2, JN.1.43.1, XBB.1.18.1, XBB.1.5 |
| Bom Jesus do Tocantins | 4 | 4 | BA.1.1, BA.1.14.1, BA.2, P.1 |
| Goiatins | 4 | 3 | JN.1.1, KP.2.3.12, XBB.1.18.1 |
| Itacajá | 4 | 3 | BA.1, JN.1.1, XDR |
| Sandolândia | 3 | 3 | B.1.1.28, P.1.7, XBB.1.18.1 |
| Itaporã do Tocantins | 3 | 2 | BA.1.14, BA.1.14.1 |
| Dois Irmãos do Tocantins | 3 | 2 | P.1, P.1.7 |
| Divinópolis Do Tocantins | 3 | 2 | BA.1.14.1, XBB.1.18.1 |
| Sucupira | 3 | 3 | BA.1.14.1, BA.5.2.1, JD.1.1 |
| Araguacu | 3 | 2 | BA.1.14, BA.1.14.1 |
| Muricilândia | 3 | 2 | AY.43, BA.1.14.1 |
| Xambioá | 3 | 2 | AY.43, AY.99.2 |
| Angico | 2 | 2 | AY.43, JN.1.43.1 |
| São Miguel Do Tocantins | 2 | 1 | AY.43 |
| Bernardo Sayão | 2 | 2 | BA.1.14.1, JN.1.29 |
| Taipas do Tocantins | 2 | 2 | BA.2.81, XBB.1.18.1 |
| Piraquê | 2 | 1 | AY.99.2 |
| Santa Rita do Tocantins | 2 | 2 | BA.1.14.1, BQ.1 |
| Santa Fé do Araguaia | 2 | 1 | BA.1.14.1 |
| Tocantinópolis | 2 | 2 | BA.5.2.1, BQ.1 |
| Filadélfia | 2 | 2 | BA.5.1, P.1 |
| Lizarda | 2 | 2 | BA.1.14.1, JN.1.43.1 |
| Fortaleza do Tabocão | 2 | 2 | BA.1.14.1, P.1.7 |
| Crixás do Tocantins | 2 | 2 | BA.1.14.1, BA.2 |
| Centenário | 2 | 2 | JN.1.1, JN.1.10 |

| | | | |
|---|---|---|---|
| Monte Santo do Tocantins | 2 | 2 | BA.1, P.1.7 |
| Oliveira ee Fátima | 2 | 2 | AY.34, XBB.1.18.1 |
| Natividade | 2 | 2 | BQ.1.1, P.1 |
| Jaú do Tocantins | 2 | 2 | BQ.1.1, P.1 |
| Juarina | 1 | 1 | BA.1.1 |
| Conceicao do Tocantins | 1 | 1 | P.1.7 |
| Couto Magalhães | 1 | 1 | AY.3 |
| Aparecida do Rio Negro | 1 | 1 | AY.99.2 |
| Araguanã | 1 | 1 | BA.1.14.1 |
| Ananás | 1 | 1 | BA.1.14.1 |
| Itaguatins | 1 | 1 | AY.43 |
| Goianorte | 1 | 1 | JN.1.61 |
| Almas | 1 | 1 | BA.5.1 |
| Tupirama | 1 | 1 | JN.1 |
| Tupiratins | 1 | 1 | AY.43 |
| Itapiratins | 1 | 1 | P.1.7 |
| Luzinópolis | 1 | 1 | JN.1 |
| Arapoema | 1 | 1 | AY.99.2 |
| Esperantina | 1 | 1 | BA.1.14.1 |
| Maurilândia do Tocantins | 1 | 1 | BA.1.14.1 |
| Cachoeirinha | 1 | 1 | AY.43 |
| Presidente Kennedy | 1 | 1 | B.1.1.28 |
| Novo Jardim | 1 | 1 | BA.2 |
| Porto Alegre Do Tocantins | 1 | 1 | BA.1.14.1 |
| Axixá do Tocantins | 1 | 1 | XBB.1.18.1 |
| Brasilândia do Tocantins | 1 | 1 | JN.1.18 |
| Palmeirante | 1 | 1 | XDR |

| | | | |
|---|---|---|---|
| Palmeiras do Tocantins | 1 | 1 | JN.2.5 |
| Bandeirantes do Tocantins | 1 | 1 | BQ.1 |
| Barcarena | 1 | 1 | BA.5.2.1 |
| Barra do Ouro | 1 | 1 | XBB.1.18.1 |
| Palmeirópolis | 1 | 1 | P.1 |

**Table S2.** Data availability for SARS-CoV-2 genomes analyzed in this study.
**GISAID Identifier**: EPI_SET_251017vr
**DOI**: https://doi.org/10.55876/gis8.251017vr
All genome sequences and associated metadata used in this study are publicly available through the GISAID EpiCoV database. Full contributor information for each sequence — including accession numbers, virus names, collection dates, originating and submitting laboratories, and author lists — can be accessed directly via dataset EPI_SET_251017vr.
**Data snapshot:**

- Total sequences: 3,961 SARS-CoV-2 genomes;
- Collection dates: 24 September 2020 to 02 September 2025;
- Geographic coverage: 1 country/territory (Brazil).
